# Hepatic WDR23 proteostasis mediates insulin homeostasis by regulating insulin-degrading enzyme capacity

**DOI:** 10.1007/s11357-024-01196-y

**Published:** 2024-05-20

**Authors:** Chatrawee Duangjan, Thalida Em Arpawong, Brett N. Spatola, Sean P. Curran

**Affiliations:** 1 Leonard Davis School of Gerontology, University of Southern California, Los Angeles, CA 90089 USA; 2 Dornsife College of Letters, Arts, and Science, University of Southern California, Los Angeles, CA 90089 USA

**Keywords:** Insulin-degrading enzyme (IDE), Insulin homeostasis, WDR23, Proteostasis, Liver, Hepatocytes, NRF2

## Abstract

Maintaining insulin homeostasis is critical for cellular and organismal metabolism. In the liver, insulin is degraded by the activity of the insulin-degrading enzyme (IDE). Here, we establish a hepatic regulatory axis for IDE through WDR23-proteostasis. *Wdr23KO* mice have increased IDE expression, reduced circulating insulin, and defective insulin responses. Genetically engineered human cell models lacking *WDR23* also increase IDE expression and display dysregulated phosphorylation of insulin signaling cascade proteins, IRS-1, AKT2, MAPK, FoxO, and mTOR, similar to cells treated with insulin, which can be mitigated by chemical inhibition of IDE. Mechanistically, the cytoprotective transcription factor NRF2, a direct target of WDR23-Cul4 proteostasis, mediates the enhanced transcriptional expression of IDE when *WDR23* is ablated. Moreover, an analysis of human genetic variation in *WDR23* across a large naturally aging human cohort in the US Health and Retirement Study reveals a significant association of *WDR23* with altered hemoglobin A1C (HbA1c) levels in older adults, supporting the use of *WDR23* as a new molecular determinant of metabolic health in humans.

**Supplementary Information:**

The online version contains supplementary material available at 10.1007/s11357-024-01196-y.

## Introduction

The incidence of diabetes continues to increase with over one million new diagnoses each year [1, 2]. Unlike cases of type I diabetes where the individual does not produce an adequate amount of insulin, individuals with type 2 diabetes (T2D) do not effectively respond to the insulin produced. More than 90% of diabetes cases are type 2, and strikingly, 90 million new cases of pre-diabetes are documented each year [1, 2]. However, it is estimated that more than 20% of individuals with diabetes are unaware of their condition. New markers linked to phenotypic outcomes are needed to improve our ability to predict T2D predisposition.

Following its secretion from Beta cells in the pancreas, clearance of endogenously released insulin is primarily achieved by hepatocytes in the liver [3]. Approximately 80% of released insulin is cleared in the first pass through the liver [4] while subsequent passage through the hepatic artery can further deplete insulin from circulation [5]. Defective insulin clearance has been linked to T2D [6] as well as hyperinsulinemia-driven systemic insulin resistance [7, 8] and hyperinsulinemia in metabolic syndrome [9]. In the obese state, hyperinsulinemia results from increased insulin secretion, but also from impaired clearance [10–12]. In addition, insulin synthesis and degradation play important roles in insulin homeostasis [4].

Insulin-degrading enzyme (IDE) is a ubiquitously expressed metalloprotease with a high affinity for insulin [6, 13]. IDE can degrade insulin in multiple intracellular compartments [14] and genetic ablation of *Ide* results in hyperinsulinemia which suggests IDE plays a central role in insulin homeostasis. However, non-proteolytic roles for IDE in insulin metabolism by downregulation of the insulin receptor have also been documented [15]. Despite these established roles in insulin metabolism, the regulatory mechanisms that govern IDE expression and activity are not fully understood.

The ubiquitin–proteasome system (UPS) is the primary protein degradation pathway, which plays an important role in cellular proteostasis [16, 17]. Proteins are targeted to the proteasome by a collection of ubiquitin-conjugating enzyme complexes, and poly-ubiquitinated target proteins are degraded by the proteasome [16]. Cullin-RING ligases (CRLs) are a well-known class of E3-ubiquitin ligases found in eukaryotes [17, 18], in which substrate receptors—including DDB1-CUL4 associated factors (DCAFs), also known as WD repeat (WDR) proteins—provide target specificity to the complex. However, the specific substrates for each receptor protein, and their functions in human health and disease, are still largely unknown. Previously, we defined WDR23 as the substrate receptor for the cytoprotective transcription factor NRF2 that functions independently to the canonical KEAP1-CUL3 regulatory pathway [19]. Moreover, GEN1 [17] and SLBP [20] are confirmed substrates of the WDR23-CUL4 proteostat, but additional substrates remain to be identified and are likely to play critical biological functions*.*

In the present study, we utilize a new *Wdr23KO* mouse model to expand upon our previous investigation of the physiological roles of WDR23, first studied in *C. elegans* [17, 19], and define a role for the WDR23 proteostasis pathway in insulin homeostasis and organismal metabolic balance. We further define human genetic variation in WDR23 as a factor associated with diabetes. Taken together, our work defines WDR23 as a new factor in cellular and organismal insulin homeostasis.

## Results

### Loss of WDR23 disrupts insulin sensitivity in male mice

**Fig. 1:**
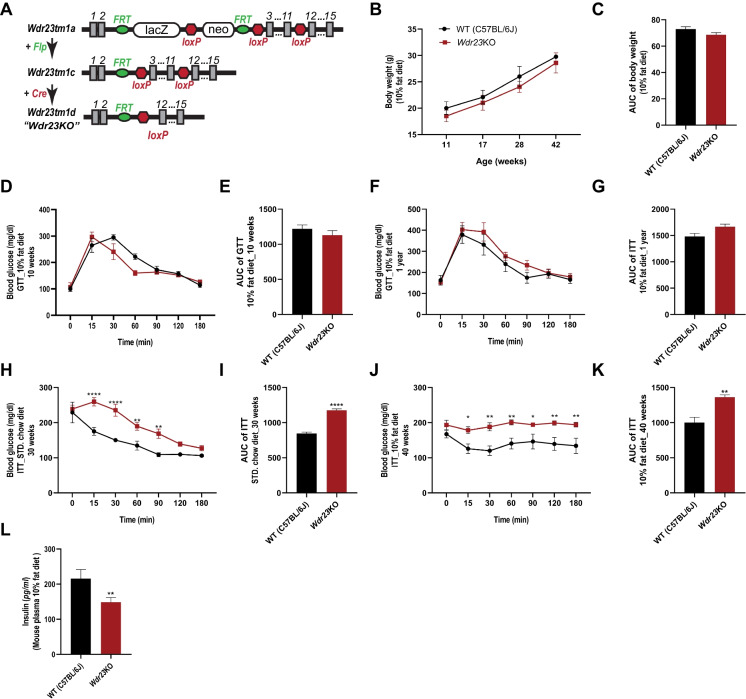
*Wdr23KO* male mice display impaired insulin homeostasis. Model of Cre-mediated germline deletion of *Wdr23* (**A**). *Wdr23KO* animals gain weight at similar rates as age-matched WT (C57BL/6J) animals (**B** and **C**). Glucose clearance, as measured by glucose tolerance testing (GTT) is similar between WT and *Wdr23KO* male mice at 10 weeks (**D** and **E**) and 1 year of age (**F** and **G**). Insulin tolerance is impaired in *Wdr23KO* male mice fed with a standard chow diet (**H** and **I**) as well as animals fed a chemically defined 10% fat diet (**J** and **K**). ELISA analysis of mouse plasma quantifying circulating insulin levels (**L**). *p < .05; **p < .01; ***p < .001; ****p < .0001

To define the role of WDR23 proteostasis in vertebrate physiology, we examined animals lacking *Wdr23* expression in all tissues [21, 22]; hereafter referred to as *Wdr23KO* (Fig. 1A). *Wdr23KO* mice are viable, display no overt defects in sexual maturity or reproductive capacity, and display normal body weight in both sexes when compared to wild-type (WT) animals over 44 weeks on a standardized 10% fat diet (Fig. 1B–C and Figure S1A-B).

We next examined the effect of *Wdr23* deletion on glucose homeostasis and insulin sensitivity by glucose tolerance test (GTT) and insulin tolerance test (ITT), respectively. At all ages tested, male *Wdr23KO* mice display an impairment of insulin sensitivity while exhibiting normal glucose tolerance when compared to age-matched WT controls (Fig. 1D–K and Figure S1C-F). However, the effects of *Wdr23KO* were sexually dimorphic as neither glucose tolerance nor insulin sensitivity were different in female *Wdr23KO* mice as compared to WT (Figure S1G-N). As such, we used male mice in all subsequent experiments.

In light of the potential differences in the responsiveness toward ectopically delivered insulin and the use of endogenously produced insulin [23], we next examined whether steady-state insulin levels were impacted by the loss of *Wdr23*. Surprisingly, we noted a significant reduction in the levels of circulating insulin in *Wdr23KO* mice (Fig. 1L).

Hepatic steatosis is associated with insulin resistance [23, 24] and as such, we next examined histological comparisons between the livers of 44-week-old mice. We observed no significant changes in the liver morphology of *Wdr23KO* mice, compared to the WT mice (Figure S1O-T). Taken together, these data indicated that the loss of *Wdr23* results in the impairment of insulin sensitivity but not glucose handling or detectable alteration of fat in the liver.

### *Wdr23KO* mice accumulate insulin-degrading enzyme (IDE) in the liver

**Fig. 2:**
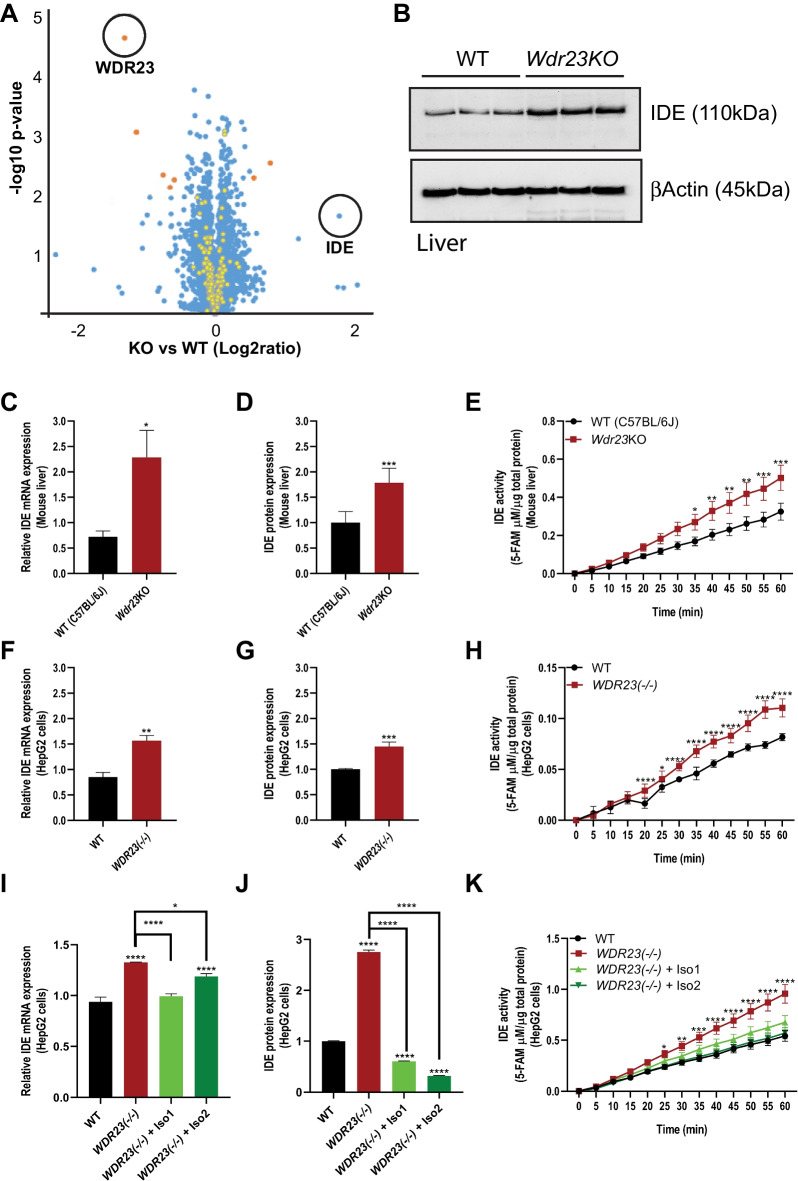
Loss of *Wdr23* increases insulin-degrading enzyme (IDE) activity in the liver. An unbiased proteomic assessment of proteins in the liver from WT and *Wdr23KO* animals reveals an increase in IDE (**A**), which is confirmed biochemically in freshly isolated livers (**B**). Increased IDE expression is increased at the mRNA (**C**) and protein (**D**) levels that result in enhanced enzymatic activity (**E**) in *Wdr23KO* livers, which is recapitulated in a HepG2 cell line with all copies of *WDR23* deleted “*Wdr23(− / −)*” (**F–H**). Rescue of *WDR23* isoforms suppresses the increased expression of IDE mRNA (**I**) and protein (**J**) and enhanced enzymatic activity (**K**) although IDE mRNA remains high in cells rescued for *WDR23* isoform 2 as compared to WT. *p < .05; **p < .01; ***p < .001; ****p < .0001

In the ubiquitin–proteasome system, the loss of a substrate receptor leads to a loss in the turnover of substrates associated with that receptor [17, 19, 25, 26]. To identify new substrates of WDR23, we performed an unbiased proteomic analysis of the liver from WT and *Wdr23KO* mice. We examined the liver as this tissue plays a crucial role in the regulation of glucose homeostasis [23] and insulin sensitivity [27] and provided adequate sample mass for analysis. A total of 209 unique proteins were identified with increased abundance across the samples; nine (9) proteins were classified as high confidence (> 30% change, *p* < 0.01), and 200 proteins were classified with moderate confidence (5–30% change, 0.01 < *p* < 0.05) (Fig. 2A; Table S3). Among the nine high-confidence proteins, we found that the level of insulin-degrading enzyme (IDE) was significantly increased in *Wdr23KO* mice liver tissue (Fig. 2A). IDE is a major enzyme responsible for insulin degradation that plays a central role in hepatic glucose metabolism [28]. As such, the increased levels of IDE could contribute to the change in insulin sensitivity observed. To support this finding, we subsequently confirmed the increased steady-state levels of IDE in fresh liver samples from age-matched WT and *Wdr23KO* mice by western blot analysis (Fig. 2B–D).

In addition, we assessed IDE proteolytic activity, which was significantly enhanced in liver homogenates from *Wdr23KO* mice as compared to WT (Fig. 2E). A similar enhancement of IDE enzymatic activity was measured in hepatocytes isolated from *Wdr23KO* mice as compared to WT hepatocytes (Figure S2A-D) suggesting a cellular defect in the major parenchymal cells of the liver [29]. To further confirm the specificity of the WDR23-dependent regulation of IDE, we developed a HepG2 cell line where we deleted all copies of *WDR23* by CRISPR/Cas9 genomic editing, hereafter called *WDR23(− / −)* (Figure S2E-F). As we observed in isolated liver tissues and primary hepatocytes, *WDR23(− / −)* HepG2 cells exhibit an increased level of steady-state IDE expression and enzymatic activity (Fig. 2F–H and Figure S2G). Importantly, transfection of HepG2 cells with GFP:*WDR23* expression plasmids [17, 19] abolished the increased IDE expression and enhanced enzymatic activity (Fig. 2I–K and Figure S2H). These data reveal that IDE expression and activity are linked to insulin metabolism defects in *Wdr23KO* mice.

### Loss of *Wdr23* interferes with the expression of the glucose metabolism pathway

**Fig. 3:**
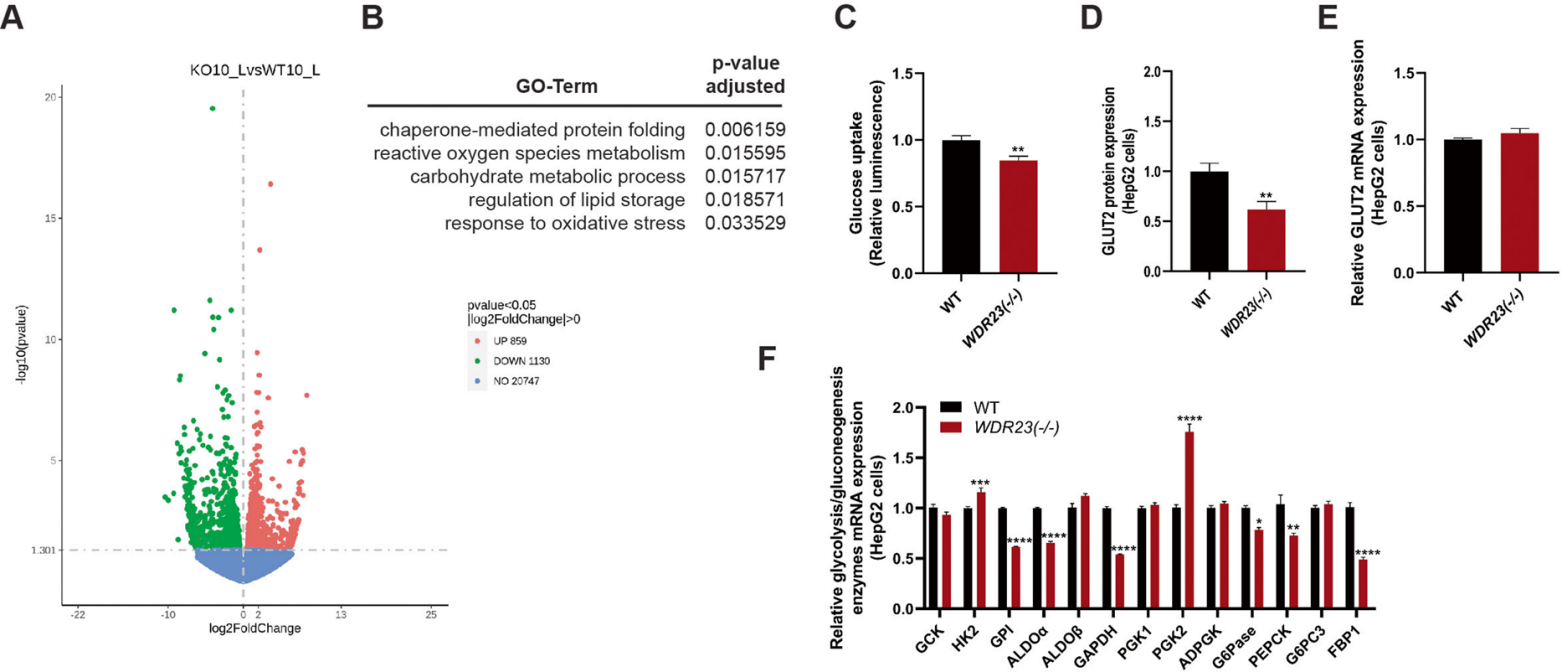
Loss of *Wdr23* disrupts glucose metabolism. RNA-sequencing analysis of male liver tissues isolated from *Wdr23KO* mice compared to WT (C57BL/6 J). Volcano plot (**A**) and lists of all differentially expressed genes (**B**) in *Wdr23*KO livers. Upregulated and downregulated genes are indicated as red and green, respectively. *Wdr23(− / −)* HepG2 cells have decreased capacity for glucose uptake (**C**) that correlated with reduced abundance of the major glucose transporter of GLUT2 protein (**D**) and mRNA (**E**) and dysregulated expression of glycolysis and gluconeogenesis enzymes (**F**). *p < .05; **p < .01; ***p < .001; ****p < .0001

Based on the altered transcriptional levels of several metabolic homeostasis genes, we next performed next-generation RNA-sequencing analyses to discern the scope of transcripts that are sensitive to the activity of the WDR23 proteostasis pathway (Fig. 3A). Significantly, pathway analysis using the GO and KEGG databases revealed the dysregulated genes induced by *Wdr23* deletion were enriched in carbohydrate metabolic processes that are regulated by insulin signaling and PPAR signaling pathways (Fig. 3B and Table S1-2). These genes are influenced by insulin secretion and signaling cascades [30, 31], which were consistent with protein expression results from both isolated liver tissue and HepG2 cells (Fig. 3B and Table S1-2). We further assessed the differentially expressed genes which revealed enrichment for components of the AGE/RAGE signaling pathway which regulates glucose metabolism in patients with diabetic complications [32] (Fig. 3B and Table S2)**.** Taken together, the loss of *Wdr23* alters the transcription of glucose and insulin metabolism pathways that result in a shift in metabolic homeostasis.

Glucose uptake by hepatocytes plays an important role in the liver’s metabolic homeostasis and its response to insulin [33, 34]. To assess whether WDR23 contributes to intracellular glucose influx, we next examined glucose absorption in HepG2 cells with and without *WDR23*. Glucose uptake was significantly decreased in *WDR23(− / −)* HepG2 cells as compared to control HepG2 cells (Fig. 3C). The major glucose transporter in the plasma membrane of hepatocytes is GLUT2 [35]. We measured the abundance of GLUT2 protein (Fig. 3D–E**)** (Figure S3A) which was significantly reduced in *WDR23(− / −)* HepG2 cells and is consistent with the reduced capacity of these cells to transport extracellular glucose. Intriguingly, SLC2A8/GLUT8, which can transport trehalose, a disaccharide consisting of two glucose molecules [36], was identified as approximately twofold upregulated in liver tissues from the *Wdr23KO* mice that may represent a compensatory response to increase carbohydrate influx (Table S4). In addition, the galactose metabolism enzyme GALE was enriched in *Wdr23KO* livers which could aid in the utilization of carbohydrate alternatives to glucose to meet cellular metabolic demands.

Based on the impaired ability of *WDR23(− / −)* HepG2 cells to transport glucose, we next measured the expression of several key enzymes in the cellular glycolysis and gluconeogenesis pathway. Consistent with a defect in glucose availability, the mRNA expression levels glycolysis and gluconeogenesis pathway genes: glucose 6-phosphatase (G6Pase), phosphoenolpyruvate carboxykinase (PEPCK), and fructose-1,6-bisphosphatase (FBP1) and glycolysis pathway genes: hexokinase 2 (HK2), glucose-6-phosphate isomerase (GPI), fructose-bisphosphate aldolase alpha (ALDOα), glyceraldehyde 3-phosphate dehydrogenase (GAPDH), and phosphoglycerate kinase 2 (PGK2) were significantly changed in *WDR23(− / −)* samples when compared with WT controls (Fig. 3F). Taken together, these data reveal a significant change in the metabolic state of hepatic cells in response to the loss of *Wdr23*.

### Loss of *Wdr23* mimics insulin treatment

**Fig. 4:**
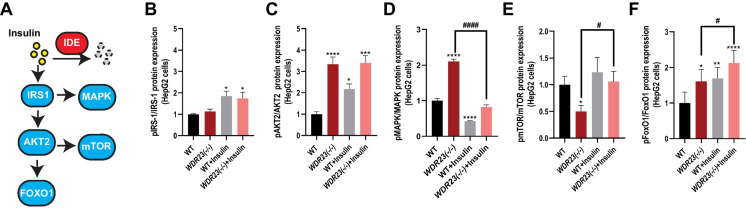
Loss of WDR23 disrupts insulin signaling. The effects of loss of *WDR2*3 in HepG2 cells, *WDR23(− / −)*, on the insulin signaling pathway (**A**) as measured by the phosphorylation state of **B** IRS1 (Tyr608/Tyr612), **C** AKT2 (Ser474), **D** MAPK (Thr202/Tyr204), **E** mTOR (Ser2448), and **F** FoxO1 (Ser256). HepG2 cells were treated with 500 nM insulin and 25 mM glucose for 24 h (+ insulin). The multiple comparisons are presented in Table S5. *p < .05; **p < .01; ***p < .001; ****p < .0001; compared to WT. ^#^*p* < .05; ^##^*p* < .01; ^###^*p* < .001; ^####^*p* < .0001 compared to *WDR23(− / −)* HepG2 cells

The insulin signaling cascade begins with insulin hormones that bind to the insulin receptor (IR), which then trigger the activation of two major kinase-dependent phosphorylation cascades through IRS1/PI3K/AKT and Ras/MAPK pathways [37]. Although there was a significant decrease in the level of circulating insulin in the *Wdr23KO* mice, which is consistent with an increased level of IDE expression that degrades insulin, we noted an increase in the phosphorylation state of several key mediators of the insulin signaling pathway (Fig. 4A). We could not detect a significant change in the phosphorylation state of IRS-1 (Fig. 4B and Figure S3B), which is phosphorylated in response to insulin binding at the insulin receptor, but phosphorylation of AKT2 in *WDR23(− / −)* HepG2 cells was increased > threefold (Fig. 4C and Figure S3C) and approximately 1.5-fold in liver homogenates from *Wdr23KO* mice (Figure S4A**)**. Similarly, phosphorylation of MAPK was increased > twofold in *WDR23(− / −)* HepG2 cells (Fig. 4D and Figure S3D) and ~ 1.5-fold increased *Wdr23KO* liver (Figure S4B). The PI3K/AKT axis of the insulin signaling cascade regulates metabolic homeostasis through multiple downstream pathways, including the forkhead transcription factor family (FoxO) and the target of rapamycin (mTOR) [31]. In *WDR23(− / −)* HepG2 cells we found a 50% decrease in the phosphorylation state of mTOR (Fig. 4E and Figure S3E) and a modest increase in the phosphorylation state of FoxO1 in *WDR23(− / −)* HepG2 cells (Fig. 4F and Figure S3F), which was similar in liver homogenates from *Wdr23KO* mice for mTOR, but not FoxO1 (Figure S4C-D**)**. We next tested whether *WDR23(− / −)* HepG2 cells are sensitive to exogenous insulin treatment. With the exception of MAPK and mTOR phosphorylation, *WDR23(− / −)* HepG2 cells mimic the phosphorylation state of WT cells treated with insulin, and treatment of *WDR23(− / −)* HepG2 cells with insulin further enhances insulin cascade phosphorylation (Fig. 4A–F and Figure S3B-F).

**Fig. 5:**
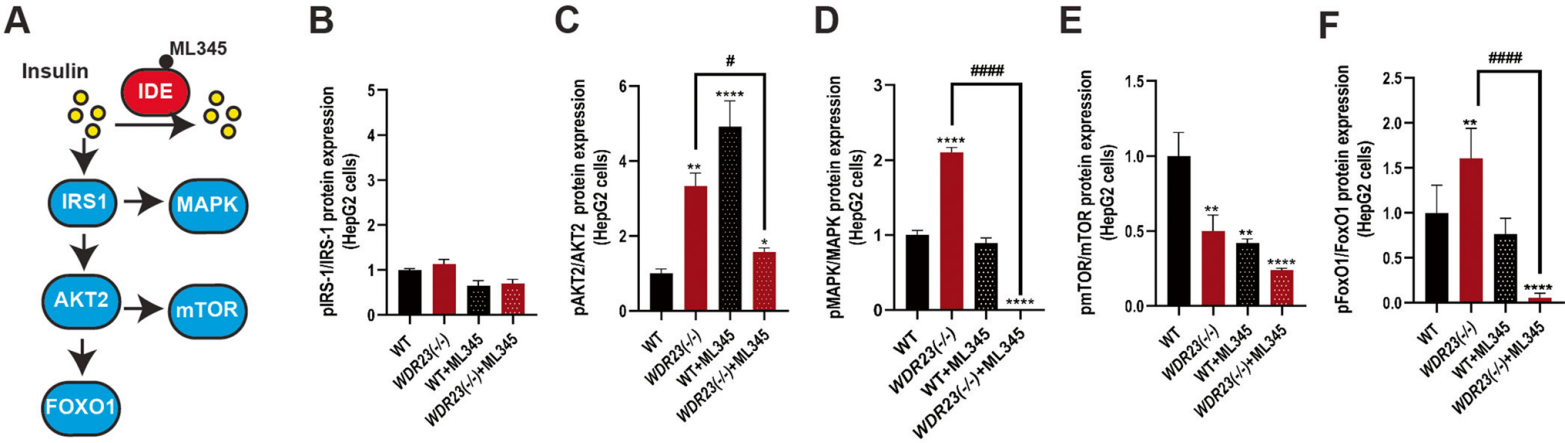
ML345 treatment reverses the effects of WDR23 loss on insulin signaling. The effects of treating WT and *WDR23(− / −)* HepG2 cells with the IDE inhibitor ML345 (40 µM, 24 h) on the insulin signaling pathway as measured by the phosphorylation state of **B** IRS1 (Tyr608/Tyr612), **C** AKT2 (Ser474), **D** MAPK (Thr202/Tyr204), **E** mTOR (Ser2448), and **F** FoxO1 (Ser256). HepG2 cells were treated with 40 µM ML345 for 24 h (+ ML345). The multiple comparisons are presented in Table S5. *p < .05; **p < .01; ***p < .001; ****p < .0001, compared to WT. ^#^*p* < .05; ^##^*p* < .01; ^###^*p* < .001; ^####^*p* < .0001, compared to *WDR23(− / −)* HepG2 cells

To link the changes in IDE expression with the changes in the phosphorylation of the insulin signaling pathway, we made use of ML345, a documented chemical inhibitor of IDE activity [38]. We first established the dose of ML345 that could effectively inhibit 50% activity in both WT and *WDR23(− / −)* HepG2 cells (Figure S5A-C) and subsequently treated cells with this concentration of inhibitor and measured the phosphorylation status of the insulin signaling pathway proteins (Fig. 5A–F). Although inhibition of IDE activity had minimal effects on IRS1 phosphorylation (Fig. 5B and Figure S5D), ML345-treated did reverse the enhanced phosphorylation of AKT2 (Fig. 5C and Figure S5E), MAPK (Fig. 5D and Figure S5F), and FoxO1 (Fig. 5F and Figure S5G) further decreased the phosphorylation of mTOR (Fig. 5E and Figure S5H). Taken together, these data support a model where the increased expression and activity of IDE in cells lacking WDR23 is causal for the defects in insulin responsiveness.

### Enhanced expression of IDE is mediated by the WDR23 substrate NRF2

We utilized ChEA3 for transcription factor enrichment analysis by orthogonal-omics integration [39] on our RNAseq data sets to identify transcription factors that mediate the responses to loss of *Wdr23* (Table S2). ChEA3 analysis revealed enrichment for several transcription factors for the 309 significantly upregulated transcripts, including CEBPB, which plays a significant role in adipogenesis, as well as in the gluconeogenic pathway [40]; CREB3L3 which plays a crucial role in the regulation of triglyceride metabolism and is required for the maintenance of normal plasma triglyceride concentrations [40, 41]; and NFE2L2/NRF2, which was expected, as our previous work identified NRF2 as a direct target substrate of WDR23 [17, 19]. NRF2 is a conserved cytoprotective transcription factor that controls the expression of stress response and intermediary metabolism gene targets [42–44]. We measured NRF2 abundance by Western blot in the liver, and similar to our findings in the murine brain [31, 45], we observed an increase in the steady-state levels of the NRF2 protein in the absence of *Wdr23* (Figure S6A-D).

**Fig. 6:**
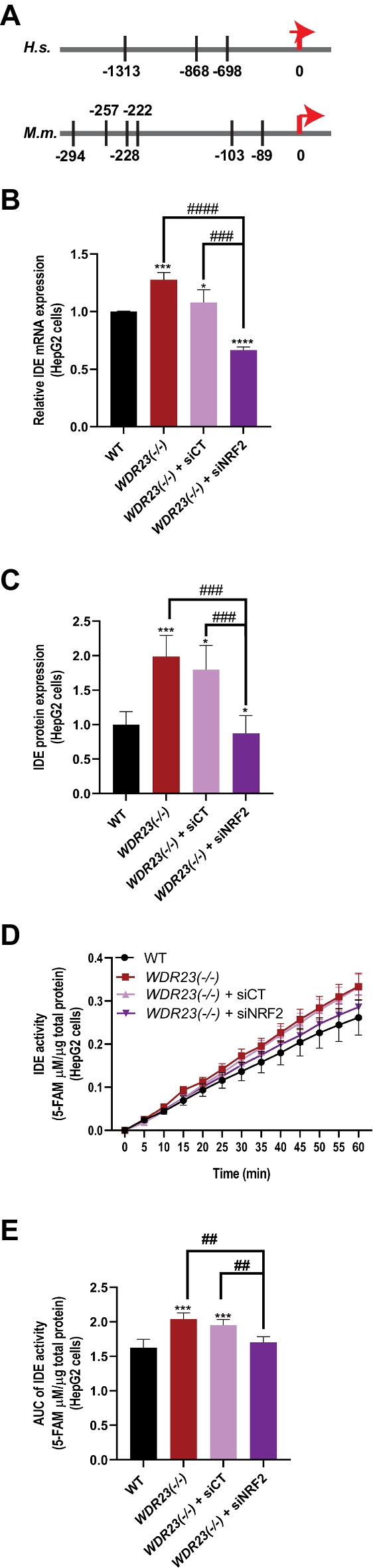
NRF2 mediates the enhanced IDE activity in response to loss of *Wdr23*. Putative ARE core binding sites in the promoter element of the human and mouse *IDE* gene (**A**); numbers are nucleotide position relative to the translational start codon (0). The cytoprotective transcription factor NRF2 is necessary for the increased expression of IDE mRNA (**B**), protein (**C**), and insulin degradation enzymatic activity (**D** and **E**). The multiple comparisons are presented in Table S5

We were curious to test whether the changes in IDE expression and the subsequent insulin metabolism phenotypes in response to loss of *Wdr23* were associated with NRF2 activation. We searched in silico within the promoter region of *Ide* for the core ARE-like NRF2 consensus binding sequence [46], 5′-TGAC-3′, and found three putative binding sites for NFE2L2/NRF2 upstream of the translational start site for the human *IDE* locus and six ARE core elements in the mouse genome [47–49] (Fig. 6A).

To confirm whether NRF2 is required for the increased expression of IDE in cells lacking *WDR23*, we examined the expression level and activity of IDE in WT and *WDR23(− / −)* HepG2 cells treated with *Nrf2*-specific siRNAs (Figure S6A). Following the transfection of *NRF2-*specific siRNA, but not the transfection with a scrambled siRNA, the enhanced expression of *IDE* transcripts (Fig. 6B), IDE protein (Fig. 6C and Figure S6E), and the enzymatic activity of IDE (Fig. 6D–E) were abrogated. These data suggest that NRF2 transcriptional activity largely contributes to the changes in insulin-degrading enzymes when the WDR23 proteostasis pathway is impaired.

### Genetic variation in *Wdr23* is associated with the incidence of diabetes in older adults

Based on the remarkable conservation in the players and responses to altered insulin signaling in metabolic health, we were curious if human *WDR23* was associated with metabolic disease states. The US Health and Retirement Study (HRS) is a nationally representative survey of adults aged 50 years and older that is an innovative tool for investigating the normal aging processes [50–52]. Recently, the HRS data sets incorporated genotypic data of participants that enable the testing of associations between normal aging phenotypes and variation across genes [53, 54].

We assessed the available phenotypic data in the US Health and Retirement Study (HRS) for SNP associations with blood-based biomarkers of diabetes and found that genetic variation in *WDR23* is associated with altered hemoglobin A1C (HbA1c) levels. HbA1c is the standard biomarker measure used for the clinical diagnosis of diabetes and pre-diabetes [55]. In HRS, multivariable linear regression models were run to test for the association between each of the five annotated *WDR23* SNPs in the HRS datasets and hemoglobin A1c measurements for each individual (Table 1), adjusting for age, gender, and principal components using PLINK. To correct for correlations between SNPs within the gene, we performed 1000 permutations, comparing shuffled (null) data to the non-shuffled data to derive the empirical *p*-value threshold of 0.0142 for HbA1c for determining statistically significant associations with *WDR23* SNPs. Taken together, our study reveals that the *WDR23* genotype (SNPs and genetic ablation) influences insulin metabolism and has the potential to impact human health during natural aging.

**Table 1:** Genetic variation in WDR23 is associated with age-related incidence of diabetes

SNP	Type	Position	Ref allele	NMISS	BETA	*P*	Significance
rs3742499	Intron variant	24586833	A	9326	− 0.015	0.1350	
rs2277481	Intron variant	24587545	G	9331	− 0.026	0.0129	*
rs17101367 (kgp6424105)	Coding variant	24587667	A	9328	− 0.012	0.2406	
rs2277482	Intron variant	24587795	A	9329	− 0.019	0.0670	#
rs2277483	Intron variant	24591892	G	9333	− 0.019	0.0650	#

HRS data association of WDR23 variants and HbA1c. HRS data association of *WDR23* variants reveals one variant with significant association with age-related diabetes as measured by HbA1c. Scan adjusted for age in 2006, sex, and four ancestral principal components

^*^p < .05; #*p* < .10; all considered suggestive with multiple test correction set at *p* = .01

## Discussion

In the present study, we reveal a role for WDR23 in the expression of IDE which suggests that targeting WDR23 could be a new biomarker for diabetes and metabolic diseases. Several lines of evidence supported the role of WDR23 in insulin sensitivity. First, loss of WDR23 disrupts insulin sensitivity in mice. Second, livers from *Wdr23*KO mice have increased IDE expression and activity. Third, the transcriptional profiling and pathway analysis indicated that genes involved in glucose and lipid metabolism, especially insulin signaling pathways were dysregulated when *Wdr23* is absent. HepG2 cells have been used as a model for insulin signaling and drug metabolism studies [56, 57], and although the metabolic activity of HepG2 cells differs from primary hepatocytes [57], an advantage of HepG2 cells that we leverage here is their sensitivity to treatment with insulin and the IDE inhibitor, ML345.

IDE is a metalloendopeptidase with a high affinity for insulin which is a major enzyme responsible for hepatic insulin degradation [28, 58]. In addition to insulin, IDE degrades glucagon, beta-amyloid peptide, and atrial natriuretic peptide [59, 60]. Impaired insulin clearance is associated with lower IDE levels that are observed in T2DM patients [28, 58]. In addition, SNPs in *Ide* locus have been linked to the risk of T2DM [61], which is similar to our finding that SNPs in *Wdr23* are associated with the risk of diabetes at older age (Table 1). Here we demonstrate that liver tissues and hepatocytes from *Wdr23KO* mice and engineered HepG2 cells lacking WDR23 have induced IDE expression and activity with a parallel decrease in circulating plasma insulin levels (Fig. 1). Correspondingly, *Wdr23KO* male mice display impaired insulin sensitivity, suggesting WDR23 is the essential regulator of insulin signaling mediated in part by the regulation of the IDE. However, there are no significant differences measured in glucose clearance and insulin tolerance in female *Wdr23*KO mice as compared to WT (Figure S1). These results are consistent with the human study by Hong et al. [62] that the variants of gene encoding IDE are strongly associated with insulin-related phenotypes in men. The insulin-dependent regulation of hepatic glucose and lipid metabolism is essential for metabolic homeostasis [63], and as such, future investigation of the impact of *Wdr23* loss on lipid metabolism and hepatic steatosis particularly on high-fat diets will be of great interest.

An imbalance in insulin signaling can drive metabolic disease due to its activity as a regulator of cellular metabolic homeostasis [31] and our unbiased proteomic analyses further revealed significant changes in key downstream mediators of the transcriptional response to insulin signaling that are central to metabolic homeostasis. Relatedly, the loss of *Wdr23* in HepG2 cells resulted in dysregulated phosphorylation of components in the insulin signaling cascade and resembled WT cells after insulin treatment (Fig. 4). However, the increased phosphorylation levels of AKT, MAPK, and FoxO were reversed in cells after treatment with the IDE inhibitor (ML345) (Fig. 5), suggesting that this change in the insulin signaling pathway in animals lacking WDR23 is associated with IDE activity.

AKT2 phosphorylation was increased in *WDR23(− / −)* HepG2 cells similar to WT cells treated with insulin (Fig. 4), but insulin treatment failed to further increase AKT2 phosphorylation suggesting an impairment of the insulin response in cells lacking *Wdr23*. Importantly, treatment with the IDE inhibitor ML345 reversed the AKT2 phosphorylation state in *WDR23(− / −)* HepG2 cells (Fig. 5), indicating the causality of increased IDE in this response. Similarly, PI3K/AKT signaling regulates metabolic homeostasis through multiple downstream pathways, including the FoxO and the mTOR [37]. We noted a modest increase in the steady-state phosphorylation of FoxO1 in *WDR23(− / −)* HepG2 cells (Fig. 4), while a decrease in *Wdr23*KO liver (Figure S4) is noted. Although the culturing conditions of the in vitro cell model likely play a role here, FoxO1 is regulated by multiple signaling pathways (e.g., EGF, glutamate, insulin, IGF1, glucose, TGFB) [2, 64–67]. These signaling pathways are critical for maintaining homeostasis and, as such, can compensate and/or act as additional layers of regulation when one pathway is activated or impaired [64]. Phosphorylated AKT can also influence mTORC1 signaling and the subsequent activation of SREBP-1, fatty acid synthase, and cholesterol-related genes [37]. The significant reduction of mTOR phosphorylation in both *WDR23(− / −)* HepG2 cells (Fig. 4) and *Wdr23*KO liver (Figure S4) further supports the activation of AKT2 signaling and reveals the importance of future work to investigate the impact of loss of *WDR23* on lipid metabolism.

The Keap1-NRF2 system is a critical target for preventing T2DM [43] as demonstrated by the activation of NRF2 through KEAP1 knockdown, which promotes glucose uptake and insulin sensitivity in diabetic mice [43, 68]. Moreover, NRF2 deletion impairs glucose handling, lipid peroxidation, and cytoprotective gene induction in mice [69] as several enzymes and proteins involved in hepatic lipogenesis and gluconeogenesis are encoded by ARE-containing NRF2 target genes [70]. Our previous work has demonstrated that WDR23 is a second mechanism for regulating NRF2 activity [17, 19], similar to the role of *C. elegans* WDR-23 on SKN-1 [17, 71–73]. Our results confirm that the increase in IDE expression in the absence of WDR23 is dependent on NRF2. As such, our study reveals a new regulatory axis of insulin homeostasis mediated by WDR23-CUL4 regulation of NRF2 and subsequent activation of IDE. Although the connection between the WDR23-mediated regulation of NRF2 by the ubiquitin–proteasome system is promising, the connection to IDE and diabetes requires further study to clarify how other WDR23 targets might influence metabolic homeostasis.

Several WDR23 SNPs were found to have a significant association with HbA1c in older adults of the HRS which is highly suggestive that the variation at this locus is linked to metabolic function in humans. However, these SNPs do not change protein-coding regions in WDR23, suggesting these SNPs could either alter *Wdr23* expression or possibly that these SNPs are marking a different effector locus linked to this region. Nevertheless, the similarities observed in our mouse model and HepG2 cell lines suggest that the WDR23 genotype is important across mammals. In the future, as the HRS genotypic data expands and becomes more diverse, an assessment of whether sex and ethnicity are significant drivers of the association between *WDR23* genotype and diabetes will be of great interest.

Collectively, our study suggests that *WDR23* plays an important role in insulin signaling and metabolic homeostasis and can provide an important data point in the development of a personalized medicine approach to ensure optimal health with age.

## Materials and methods

### Animals

All animal protocols were approved by the Institutional Animal Care and Use Committee (IACUC) of the University of Southern California, and all the procedures were conducted in compliance with institutional guidelines and protocols.

*Wdr23* knock-out (*Wdr23*KO) (Figure S2I) mice were generated by Wellcome Trust Sanger Institute [74, 75]. *Wdr23KO* animals were subsequently backcrossed nine times into our C57BL/6 J (WT) strain from the Jackson laboratory. Heterozygous (*Wdr23* + */ −*) *dams* and *sires* were then mated to generate *Wdr23* + */* + and *Wdr23 − / − *animals that were maintained as WT and KO, respectively. Male and female mice (*n* = 4–6/group) were kept in a 12:12-h light–dark cycle, constant temperature, and humidity room. All animals were allowed ad libitum access to water and food.

### Rodent diets

Mice were fed ad libitum with irradiated 5053 rodent chow diet (Lab Diet) containing 3.4 kcal/g (23.6% protein, 64.5% carbohydrate, and 11.9% fat) or D12450B rodent diet with 10 kcal% fat (research diet), containing 3.9 kcal/g (20.0 % protein, 70.0% carbohydrate, and10.0% fat).

### Glucose tolerance tests (GTT)

Performed as previously described [76], animals (10, 17, and 28 weeks and 1 year old) were fasted for 18 h prior to administration of glucose (2 g/kg body weight) via intraperitoneal injection. Blood glucose was measured from the tail tip at 0, 15, 30, 60-, 90-, 120-, and 180-min post-injection (Contour Next One Blood Glucose monitoring (9763)).

### Insulin tolerance tests (ITT)

Performed as previously described [76], animals (30- and 40-week-old mice) were fasted for 4 h and then injected intraperitoneally with recombinant human insulin at (0.5 U/kg bodyweight), and glucose levels were determined at 0, 15, 30, 60, 90, 120, and 180 min after insulin injection.

### IDE activity assay

The enzymatic activity of IDE in mice liver tissue and HepG2 cells was determined using Sensolyte 520 IDE Activity Assay fluorometric kit according to the manufacturer’s protocol.

### Determination of plasma hormones

Animals were anesthetized, blood samples were taken and centrifuged for 10 min at 10,000 rpm, and the supernatant (plasma) was used for hormone measurement. Plasma insulin levels were measured using an ELISA kit (Meso Scale Discovery) according to the manufacturer’s instructions.

### RNA extraction and real-time quantitative PCR

Quantitative PCR was performed as previously described [19]. Briefly, mice liver tissue or HepG2 cells were collected and lysed in Tri reagent (Zymo research). RNA was extracted according to the manufacturer’s protocol. RNA was reverse-transcribed to complementary DNA using the qScript cDNA SuperMix (Quanta Biosciences). Quantitative PCR was conducted by using the SYBR Green (BioRad). The relative expression of each gene was normalized against the internal control gene (GAPDH), and expression levels were analyzed using the 2-ΔΔCT method. The gene-specific sequences of the primers for HepG2 cells and mouse liver tissue are presented in the key resources table.

### RNA-seq

Isolated RNA was sent to Novogene for library preparation and deep sequencing in biological triplicate. The read counts were used for differential expression (DE) analysis by using the R package DEseq2 (R version 3.5.2). Differentiated expressed genes were analyzed using *p*-value < 0.05 and fold change > 1.5 as the cutoff.

### Western blot analysis

Whole-cell lysates were prepared in M-PER buffer (1 × Mammalian Protein Extraction Reagent (Thermo Scientific), 0.1% Halt Protease & Phosphatase inhibitor (Thermo Scientific) according to the manufacturer’s protocol. Total protein concentrations were quantified by Bradford assay (Sigma). An equal amount of protein (20 µg) was separated on 4–12% bis–tris polyacrylamide gel (Invitrogen) in MOPS running buffer (Invitrogen) and then transferred to nitrocellulose membranes (GE Healthcare Life Science). After blocking for 1 h with 3% BSA in PBST (PBS, 0.1% Tween 20), the membranes were subjected to immunoblot analysis. Antibodies used include IDE (Millipore sigma, 1:5000), IRS-1 (Cell Signaling Technology, 1:500), pIRS-1 (Millipore Sigma, 1:500), AKT2 (Cell Signaling Technology, 1:1000), pAKT2 (Cell Signaling Technology, 1:1000), MAPK (Cell Signaling Technology, 1:1000), pMAPK (Cell Signaling Technology, 1:1000), mTOR (Cell Signaling Technology, 1:1000), pmTOR (Cell Signaling Technology, 1:1000), FoxO1 (Cell Signaling Technology, 1:1000), pFoxO1 (Cell Signaling Technology, 1:1000), β-actin (Millipore Sigma, 1:10,000), and HRP-conjugated secondary antibodies (Thermo Fisher, 1:10,000). Specific protein bands were visualized and evaluated using FluorChem HD2 (ProteinSimple). The full images of electrophoretic blots are presented in supplementary materials.

### Isolation of mouse primary hepatocytes

Mouse primary hepatocytes were isolated from male *Wdr23*KO and WT mice, aged 3–4 weeks, using modified collagenase perfusion methods. Briefly, the liver was perfused via the portal vein with perfusion medium (GIBCO) for 6 min and liver digest medium (GIBCO) for 5 min. The liver was removed and placed in a 100-mm plate filled with cold washing medium (Williams’ E medium (WEM, GIBCO), supplemented with primary hepatocyte maintenance supplements (GIBCO). The liver was dispersed into small pieces in the medium using forceps and filtered through a 100/70-µM cell strainer into a falcon tube. Cells are collected by centrifugation at 50 g for 3 min and washed 3 times with 20 ml washing medium. The cells were counted, and viability was evaluated by trypan blue exclusion. Hepatocytes were plated in 6-well plates (pre-coated with 0.01% collagen in acetic acid (Sigma) at a density of 2 × 10^5^ cells per well in maintenance medium (WEM, GIBCO), supplemented with primary hepatocyte maintenance supplements (GIBCO) and incubated for 2–3 h, 37 °C, 5% CO_2_.

### Cell culture and transfections

WDR23-depleted (*WDR23(− / −)*) HepG2 cells were generated by CRISPR/Cas9 (Synthego). Cells were maintained in Minimum Essential Medium (GIBCO) supplemented with 10% fetal bovine serum (GIBCO) and 1% antibiotic/antimycotic (Corning) at 37 °C, 5% CO_2_.

Full-length cDNA sequences of Hs *WDR23* Isoforms 1 and 2 were cloned into pcDNA 6.2/N-EmGFP/TOPO (Thermo Fisher), as previously described [19]. siRNAs (Thermo Fisher) used include NRF2 (s9492) and control no.1 (4390843).

Transfections were performed with Lipofectamine 3000 (Thermo Fisher) and Lipofectamine RNAiMAX (Thermo Fisher) according to the manufacturer’s protocol.

For the establishment of insulin resistance in HepG2 cells, cells were seeded in 6-well plates with normal medium overnight. After reaching 80% confluence, the medium was replaced with SF-MEM and incubated for 24 h. Subsequently, the cells were treated with SF-MEM supplemented with 500 nM insulin and 25 mM glucose for 24 h.

### Glucose uptake measurements

Glucose uptake was performed by using Glucose Uptake-Glo from Promega. Briefly, HepG2 cells were seeded in 96-well plates (2 × 10^4^ cells/well) for 24 h. *WDR23(− / −)* HepG2 cells were transiently transfected with indicated plasmids for 24 h. Samples were prepared in triplicates for Glucose Uptake-Glo according to the manufacturer’s protocol, and another setup was for cell viability assay to normalize the cell number.

### Cell viability

MTT was used to measure cell viability. A total of 0.5 mg/ml MTT was added to the culture medium and incubated for 3 h at 37 °C. The formazan crystals were solubilized by DMSO. The absorbance (550) or luminescence was measured using a SPECTRA max M2 Plate Reader. Intracellular glucose uptake was expressed as relative luminescence which was normalized by cell viability.

### Histological analysis

Liver sections were stained with hematoxylin and eosin (H&E) to visualize adipocytes and inflammatory cells in the tissues. Sections and cells were analyzed by Thunder Imaging Leica DMi8 microscope. H&E-stained sections (six slides for each sample) were randomly selected and quantified for the steatosis area using the Fiji ImageJ-win64 (Max Planck Institute of Molecular Cell Biology and Genetics, Dresden Germany).

### Protein mass spectroscopy

Proteomic characterization of the proteome of mice liver tissues was performed by Poochon Scientific. Briefly, the total protein extractions of liver tissue samples (44 weeks) were prepared following Poochon SOP#602 protocols. The protein concentration of the supernatants was determined by the BCA protein assay kit. A total of 90 µg of protein lysate from each sample was run on SDS-PAGE followed by in-gel trypsin digestion, TMT-10plex labeling, and LC/MS/MS analysis. The LC/MS/MS analysis was carried out using a Thermo Scientific Q-Exactive hybrid Quadrupole-Orbitrap Mass Spectrometer and Thermo Dionex UltiMate 3000 RSLCnano System. Each peptide fraction was loaded onto a peptide trap cartridge at a flow rate of 5 µl/min. The trapped peptides were eluted onto a reversed-phase 20 cm C18 PicoFrit column (New Objective, Woburn, MA) using a linear gradient of acetonitrile (3–36%) in 0.1% formic acid, for 100 min at a flow rate of 0.3 µl/min. Then, the eluted peptides from the column were ionized and sprayed into the mass spectrometer, using a Nanospray Flex Ion Source ES071 (Thermo) under the following settings: spray voltage, 1.8 kV and capillary temperature, 250 °C. MS Raw data files were searched against the human protein sequence database or other species protein sequence database obtained from the NCBI website using the Proteome Discoverer 1.4 software (Thermo, San Jose, CA) based on the SEQUEST and percolator algorithms. The false positive discovery rates (FDR) were set at 1%. The resulting Proteome Discoverer Report contains all assembled proteins with peptide sequences and peptide spectrum match counts (PSM#) and TMT-tag-based quantification ratio. TMT-tag-based quantification was used to determine the relative abundance of proteins identified in each set of samples. The calculation and statistical analysis use Microsoft Excel functions. The heat map was generated using R. The annotation including pathways and processes was based on the Kyoto Encyclopedia of Genes and Genomes (KEGG) pathway database, the UniProtKB protein database, and the NCBI protein database. Samples were normalized to 353 proteins, which were used as control, due to no change between WT and *Wdr23KO* liver samples.

### HRS GeneWAS, population stratification, regression models and other covariates, and SNP evaluation

In brief, the US HRS [51, 52, 54] is a nationally representative, longitudinal sample of adults aged 50 years and older, who have been interviewed every 2 years, beginning in 1992. Because the HRS is nationally representative, including households across the country and the surveyed sample now includes over 36,000 participants, it is often used to calculate national prevalence rates for specific conditions for older adults, including physical and mental health outcomes, cognitive outcomes, as well as financial and social indicators. The sample for the current study is comprised of a subset of the HRS for which genetic data were collected, as described below. To reduce potential issues with population stratification, the GeneWAS in this study was limited to individuals of primarily European ancestry. The final sample was *n* = 3319, with the proportion of women at 58.5%.

#### HRS Participants

Data are from the *Health and Retirement Study* (HRS), a nationally representative sample of older Americans aged 50 and over [77, 78] in the contiguous United States. The present analysis was limited to participants who self-reported their race as white/Caucasian, verified by principal components analysis of ancestry markers, in order to assess the effects of DCAF11 variation found in European ancestry groups. The analytical sample for the HRS included individuals who had available genetic data, at least one measure of hemoglobin A1C data, and relevant covariate data (*n* = 9326 to 9333 per SNP based on sample and SNP quality).

#### DCAF11 single nucleotide polymorphisms (SNPs)

For HRS, genotype data were accessed from the National Center for Biotechnology Information Genotypes and Phenotypes Database (dbGaP [79]). Genotyping was conducted on over 15,000 individuals using the Illumina HumanOmni2.5-4v1 (2006 and 2008) and HumanOmni2.5-8v1 (2010) arrays and was performed by the NIH Center for Inherited Disease Research (CIDR). Standard quality control procedures were implemented by the University of Washington Genetic Coordinating Center [80]. Further detail is provided in HRS documentation [81]. The DCAF11 SNPs were filtered to include only those with a minor allele frequency of 5% or greater. SNPs were coded in order to assess the additive effects of each additional allele (i.e., 0, 1, or 2 minor alleles) and were extracted using PLINK 1.9 [82, 83].

#### Hemoglobin A1C biomarkers

The HRS collected biomarkers from blood spots, including glycosylated hemoglobin (HbA1c), which is an indicator of glycemic control over the past 2–3 months. HbA1c was available from blood spots on half of the sample in 2006, and the other half in 2008, with additional individuals captured in the 2010 or 2012 data collection waves. Detailed information on collection and assay are provided elsewhere [84, 85].

#### Covariates

In HRS, covariates included age at biomarker assessment, gender (0 = female, 1 = male), and four principal components to reduce such type 1 error due to differences in underlying population substructure [86, 87]. Detailed descriptions of the processes employed for running principal components analysis, including SNP selection, are provided by HRS [81], and follow methods outlined by Patterson and colleagues [88].

#### Statistical analysis

All experiments were performed at least in triplicate. Data are presented as mean ± SEM. Data handling and statistical processing were performed using GraphPad Prism 8.0. Comparisons between the two groups were done using an unpaired Student’s *t*-test. Comparisons between more than two groups were done using one-way ANOVA. Differences were considered significant at the *p* ≤ 0.05 level.

In HRS, multivariable linear regression models were run to test for the association between each of the five DCAF11 SNPs and hemoglobin A1C, adjusting for age, gender, and principal components using PLINK. With the number of SNPs and primary phenotypes in this study, strict Bonferroni correction would yield an adjusted multiple test-correction *p*-value threshold of 0.01 (for 5 SNP tests). However, a Bonferroni correction is too conservative for this type of gene-level assessment because of the correlations between SNPs within the gene [89, 90]. To address this, we calculate empirical *p*-value thresholds, through permutation procedures [89–92]. Permutation is a process whereby the correlations between SNPs and phenotypes are intentionally shuffled and then *p*-values calculated for the shuffled (null) data are compared to the non-shuffled data. This procedure is repeated multiple times in order to determine an empirical *p*-value [90, 92, 93], an empirically derived threshold at which a test result is less likely to achieve significance by chance alone. We performed 1000 permutations using PLINK to derive the empirical *p*-value threshold of 0.0142 for HbA1c for determining statistically significant associations with DCAF11 SNPs.

### Supplementary Information

Below is the link to the electronic supplementary material.

Supplementary file1 (JPG 4613 KB)

Supplementary file2 (JPG 1634 KB)

Supplementary file3 (JPG 1586 KB)

Supplementary file4 (JPG 2199 KB)

Supplementary file5 (JPG 2561 KB)

Supplementary file6 (JPG 1331 KB)

Supplementary file7 (DOCX 17 KB)

Supplementary file8 (DOCX 15 KB)

Supplementary file9 (DOCX 17 KB)

Supplementary file10 (DOCX 31 KB)

Supplementary file11 (DOCX 16 KB)

## Data Availability

The datasets are produced by the University of Michigan, Ann Arbor. The HRS phenotypic data files are public-use datasets, available at https://hrs.isr.umich.edu/data-products/access-to-public-data. The HRS genotype data are available to authorized researchers: https://www.ncbi.nlm.nih.gov/projects/gap/cgi-bin/study.cgi?study_id=phs000428.v2.p2https://www.ncbi.nlm.nih.gov/projects/gap/cgi-bin/study.cgi?study_id=phs000428.v2.p2
