# Supplementary figures and images for: Hepatic WDR23 proteostasis mediates insulin homeostasis by regulating insulin-degrading enzyme capacity
Source: GeroScience. 2024 May 20;46(5):4461–78. doi: 10.1007/s11357-024-01196-y (PMC11336002; doi:10.1007/s11357-024-01196-y)

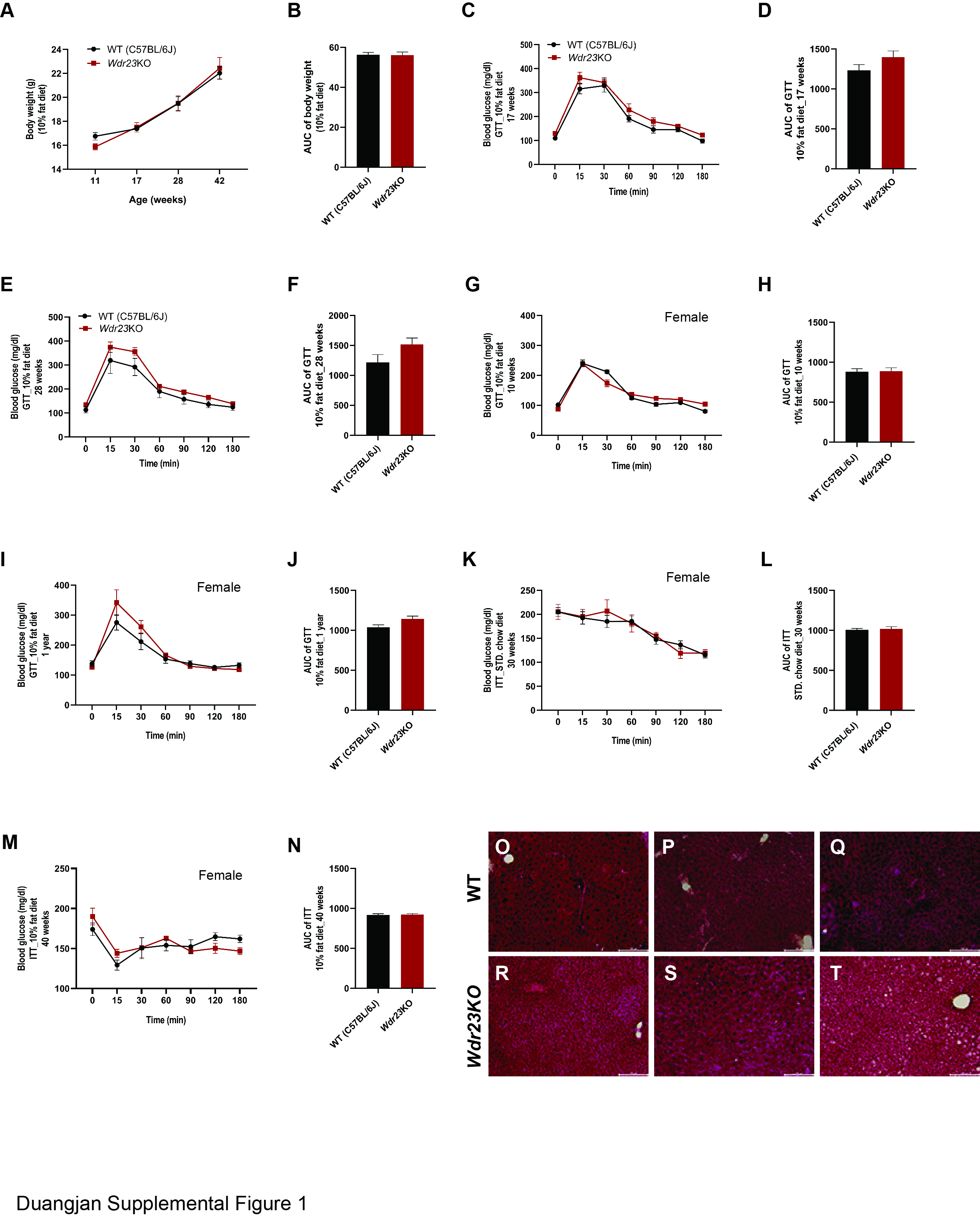

Supplement: Supplementary file 1 — Supplementary file1 (JPG 4613 KB) [file 11357_2024_1196_MOESM1_ESM.jpg]

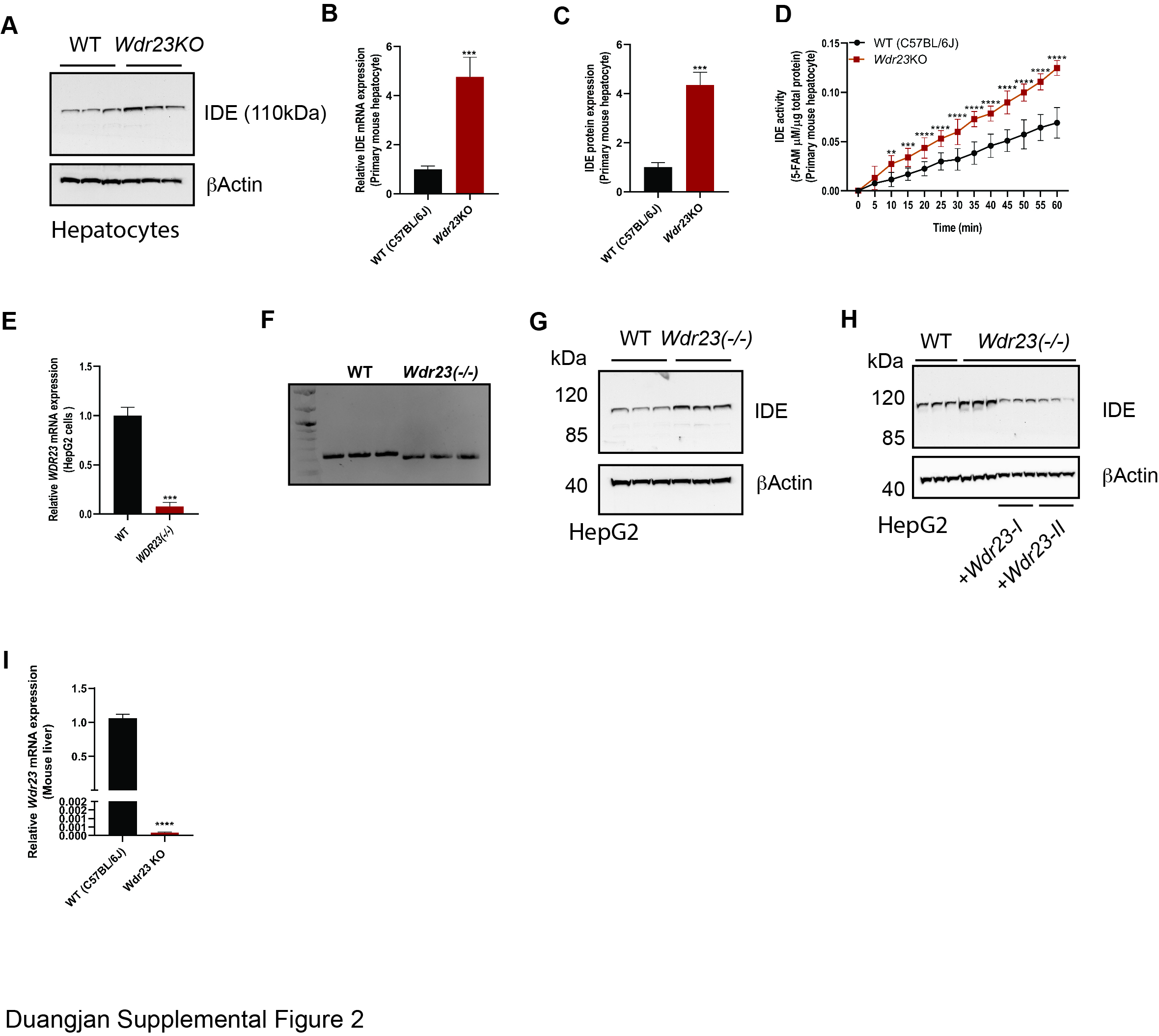

Supplement: Supplementary file 2 — Supplementary file2 (JPG 1634 KB) [file 11357_2024_1196_MOESM2_ESM.jpg]

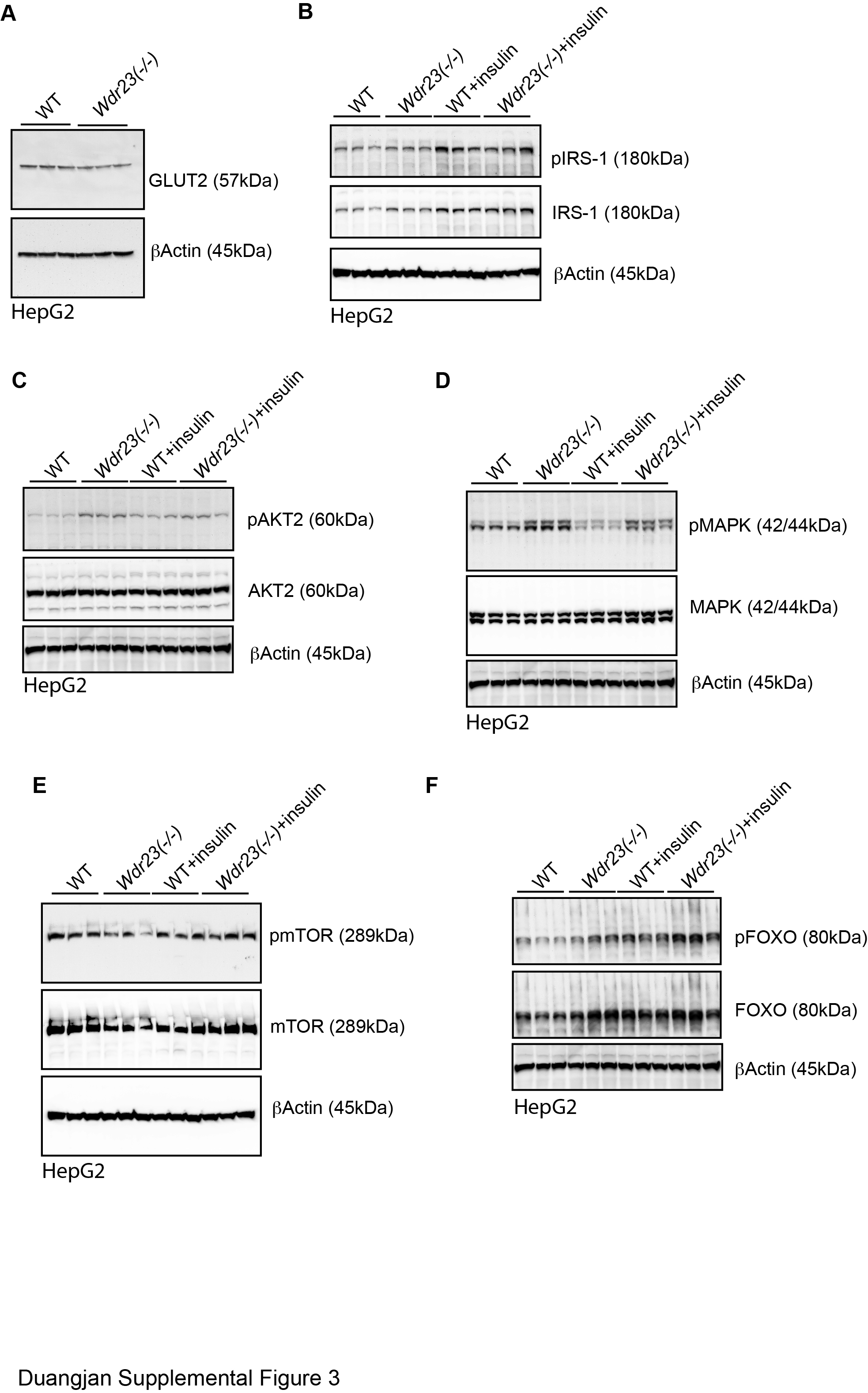

Supplement: Supplementary file 3 — Supplementary file3 (JPG 1586 KB) [file 11357_2024_1196_MOESM3_ESM.jpg]

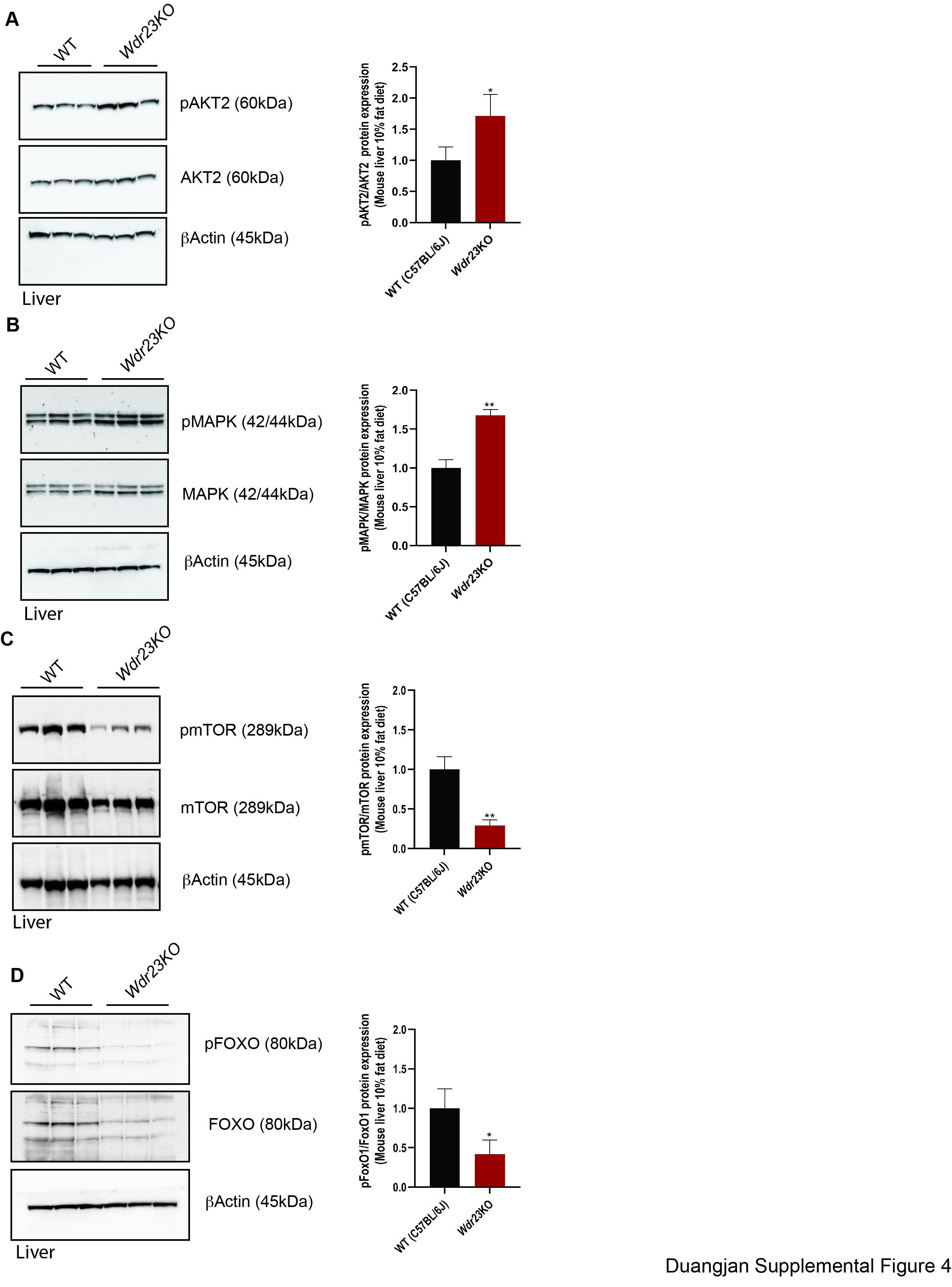

Supplement: Supplementary file 4 — Supplementary file4 (JPG 2199 KB) [file 11357_2024_1196_MOESM4_ESM.jpg]

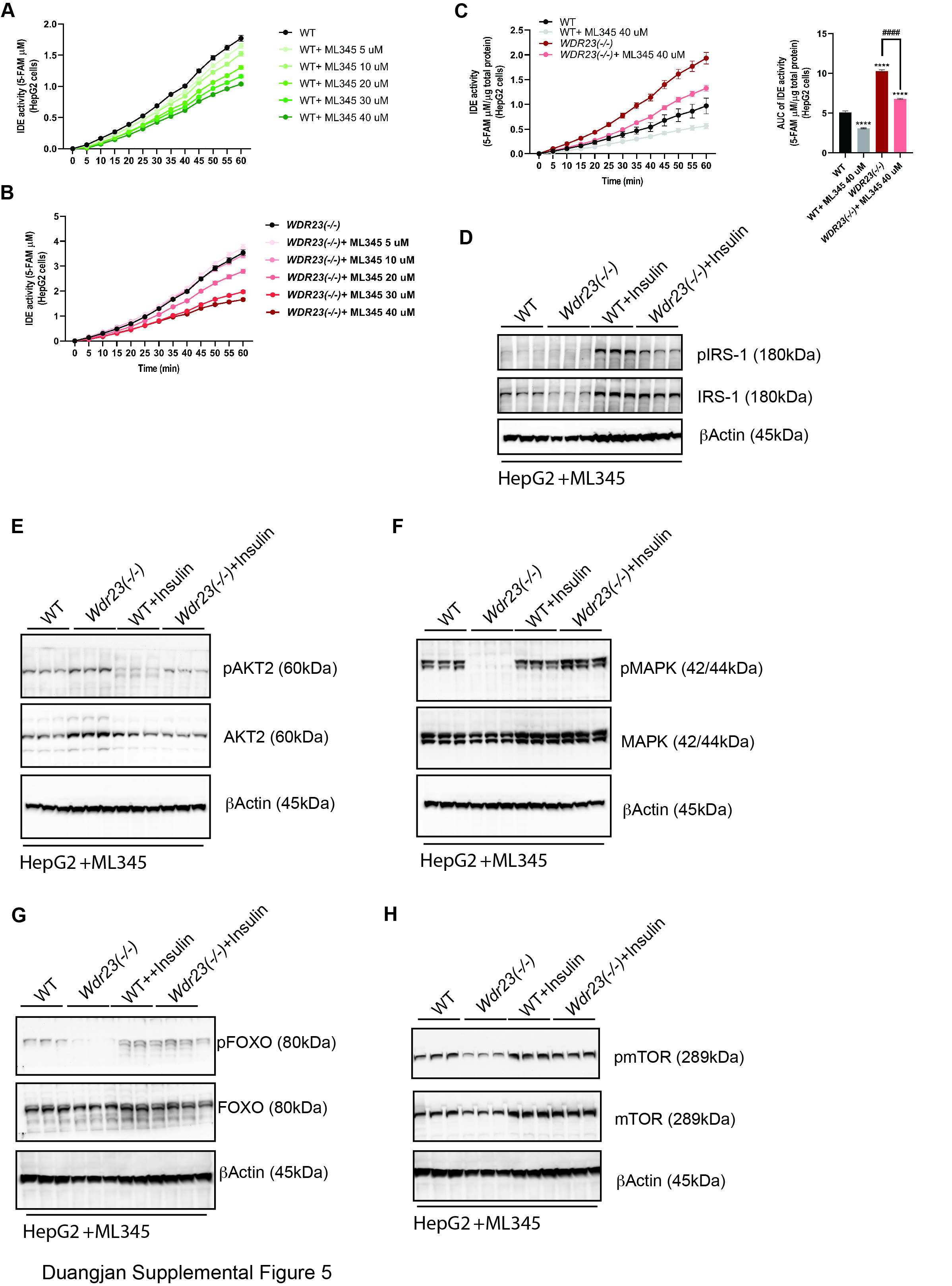

Supplement: Supplementary file 5 — Supplementary file5 (JPG 2561 KB) [file 11357_2024_1196_MOESM5_ESM.jpg]

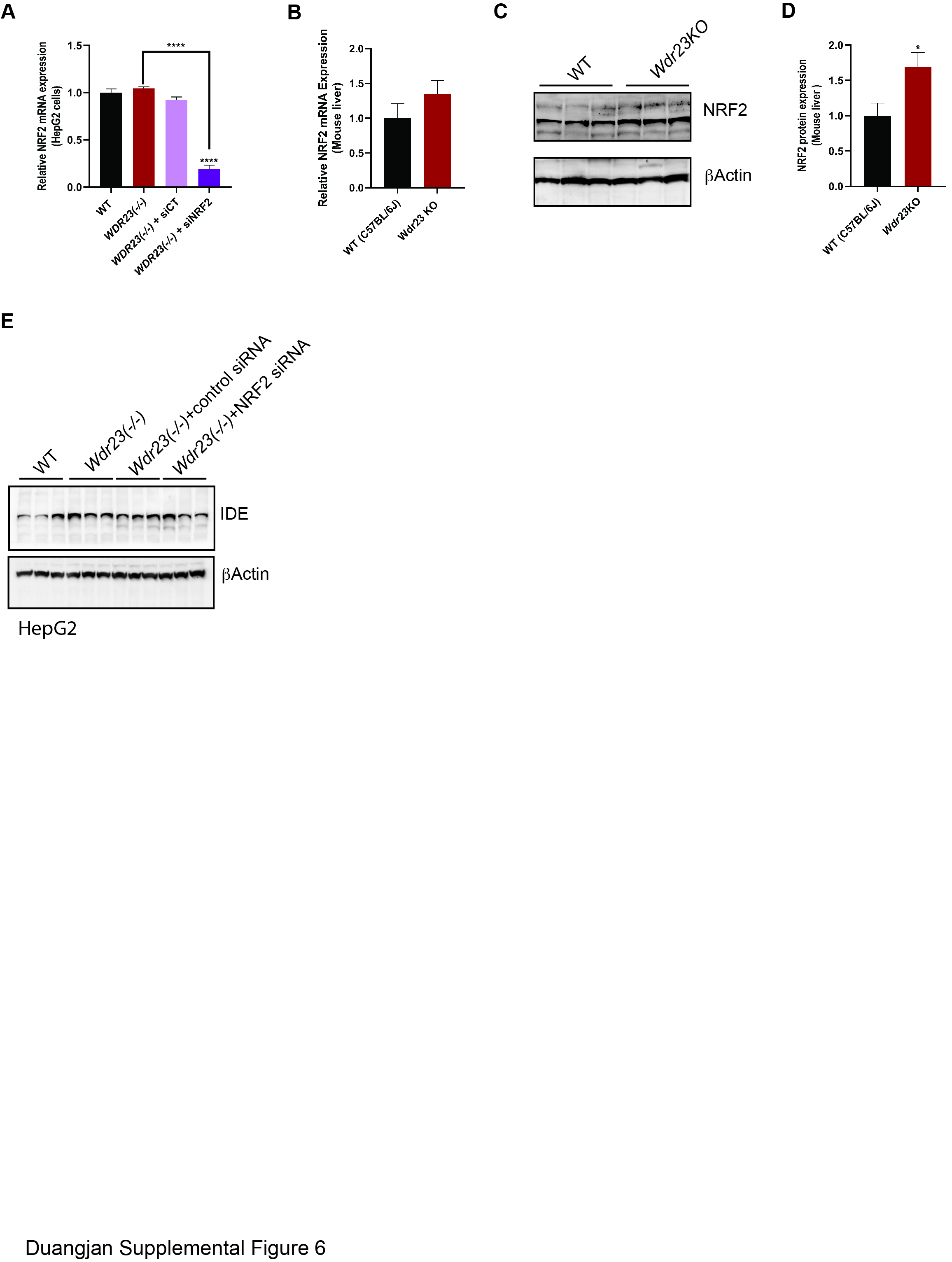

Supplement: Supplementary file 6 — Supplementary file6 (JPG 1331 KB) [file 11357_2024_1196_MOESM6_ESM.jpg]
