# Supplementary material for: Hepatic WDR23 proteostasis mediates insulin homeostasis by regulating insulin-degrading enzyme capacity
Source: GeroScience. 2024 May 20;46(5):4461–78. doi: 10.1007/s11357-024-01196-y (PMC11336002; doi:10.1007/s11357-024-01196-y)
Supplement: Supplementary file 7 — Supplementary file7 (DOCX 17 KB) [file 11357_2024_1196_MOESM7_ESM.docx]

**Table S1. The GO functional enrichment analysis of DEGs in *Wdr23*KO mice liver tissues compare to the WT control with the threshold of *P*≤0.05**

| **Term** | **Gene ID** | **Chr** | **Name** | **Description** | **Log2 Fold Change** | ***P*-value** | ***P*-adj** |
| --- | --- | --- | --- | --- | --- | --- | --- |
| **Carbohydrate metabolic process**  **Up-regulated** | | | | | | | |
|  | ENSMUSG00000041237 | 3 | Pklr | Pyruvate kinase liver and red blood cell | 1.842783 | 3.55E-10 | 5.68E-07 |
|  | ENSMUSG00000004815 | 5 | Dgkq | Diacylglycerol kinase | 2.009794 | 1.57E-08 | 1.33E-05 |
|  | ENSMUSG00000041798 | 11 | Gck | Glucokinase | 1.839161 | 5.97E-06 | 0.00141 |
|  | ENSMUSG00000025815 | 2 | Dhtkd1 | Dehydrogenase E1 and transketolase domain containing 1 | 1.114049 | 7.15E-05 | 0.008823 |
|  | ENSMUSG00000029802 | 6 | Abcg2 | ATP binding cassette subfamily G member 2 (Junior blood group) | 1.101674 | 0.000264 | 0.021585 |
|  | ENSMUSG00000034793 | 11 | G6pc3 | Glucose 6 phosphatase, catalytic, 3 | 1.298063 | 0.000421 | 0.030001 |
|  | ENSMUSG00000025236 | 9 | Adpgk | ADP-dependent glucokinase | 1.300949 | 0.00093 | 0.048996 |
|  | ENSMUSG00000041237 | 3 | Pklr | Pyruvate kinase liver and red blood cell | 1.842783 | 3.55E-10 | 5.68E-07 |
|  | ENSMUSG00000025815 | 2 | Dhtkd1 | Dehydrogenase E1 and transketolase domain containing 1 | 1.114049 | 7.15E-05 | 0.008823 |
|  | ENSMUSG00000032310 | 9 | Cyp1a2 | Cytochrome P450, family 1, subfamily a, polypeptide 2 | 1.524902 | 0.000215 | 0.018769 |
|  | ENSMUSG00000025153 | 11 | Fasn | Fatty acid synthase | 1.817651 | 0.000268 | 0.021806 |
|  | ENSMUSG00000030972 | 7 | Acsm5 | Acyl-CoA synthetase medium-chain family member 5 | 1.005508 | 0.000429 | 0.030242 |
|  | ENSMUSG00000025236 | 9 | Adpgk | ADP-dependent glucokinase | 1.300949 | 0.00093 | 0.048996 |
| **Carbohydrate metabolic process**  **Down-regulated** | | | | | | | |
|  | ENSMUSG00000000628 | 6 | Hk2 | Hexokinase 2 | -3.22647 | 5.12E-06 | 0.001263 |
|  | ENSMUSG00000024042 | 17 | Sik1 | salt inducible kinase 1 | -1.72827 | 1.38E-05 | 0.002671 |
|  | ENSMUSG00000025190 | 19 | Got1 | Glutamic-oxaloacetic transaminase 1 | -1.30683 | 7.23E-05 | 0.008859 |
|  | ENSMUSG00000060402 | 7 | Chst8 | Carbohydrate (N-acetylgalactosamine 4-0) sulfotransferase 8 | -7.0051 | 0.000148 | 0.014403 |
|  | ENSMUSG00000024029 | 17 | Tff3 | Trefoil factor 3, intestinal | -7.60111 | 0.000173 | 0.015887 |
|  | ENSMUSG00000038155 | 19 | Gstp2 | Glutathione S-transferase, pi 2 | -3.93441 | 3.93E-11 | 7.12E-08 |
|  | ENSMUSG00000030895 | 7 | Hpx | Hemopexin | -1.05529 | 7.74E-06 | 0.001801 |
|  | ENSMUSG00000018339 | 11 | Gpx3 | Glutathione peroxidase 3 | -1.63836 | 0.000341 | 0.025717 |
|  | ENSMUSG00000031584 | 8 | Gsr | Glutathione reductase | -1.10922 | 0.000844 | 0.046274 |
| **Fatty acid metabolic process**  **Up-regulated** | | | | | | | |
|  | ENSMUSG00000020538 | 11 | Srebf1 | Sterol regulatory element binding transcription factor 1 | 2.246853 | 2.76E-07 | 0.000145 |
|  | ENSMUSG00000038754 | 19 | Elovl3 | Elongation of very long chain fatty acids (FEN1/Elo2, SUR4/Elo3, yeast)-like 3 | 1.941961 | 6.96E-07 | 0.000298 |
|  | ENSMUSG00000054422 | 6 | Fabp1 | Fatty acid binding protein 1, liver | 1.256687 | 0.000107 | 0.011728 |
|  | ENSMUSG00000025153 | 11 | Fasn | Fatty acid synthase | 1.817651 | 0.000268 | 0.021806 |
|  | ENSMUSG00000030972 | 7 | Acsm5 | Acyl-CoA synthetase medium-chain family member 5 | 1.005508 | 0.000429 | 0.030242 |
|  | ENSMUSG00000010651 | 9 | Acaa1b | Acetyl-Coenzyme A acyltransferase 1B | 0.952087 | 0.000938 | 0.049223 |
|  | ENSMUSG00000041798 | 11 | Gck | Glucokinase | 1.839161 | 5.97E-06 | 0.00141 |
|  | ENSMUSG00000040505 | 17 | Abcg5 | ATP binding cassette subfamily G member 5 | 1.47019 | 0.00028 | 0.022564 |
|  | ENSMUSG00000047822 | 9 | Angptl8 | Angiopoietin-like 8 | 1.571686 | 0.000583 | 0.036294 |
|  | ENSMUSG00000005677 | 1 | Nr1i3 | Nuclear receptor subfamily 1, group I, member 3 | 2.030792 | 0.000657 | 0.03918 |
| **Fatty acid metabolic process**  **Down-regulated** | | | | | | | |
|  | ENSMUSG00000039202 | 7 | Abhd2 | Abhydrolase domain containing 2 | -2.58157 | 1.2E-05 | 0.002434 |
|  | ENSMUSG00000015568 | 8 | Lpl | Lipoprotein lipase | -2.73116 | 1.56E-05 | 0.002854 |
|  | ENSMUSG00000074254 | 7 | Cyp2a4 | Cytochrome P450, family 2, subfamily a, polypeptide 4 | -7.31411 | 0.000103 | 0.0116 |
|  | ENSMUSG00000028341 | 4 | Nr4a3 | Nuclear receptor subfamily 4, group A, member 3 | -7.15208 | 0.000104 | 0.01162 |
|  | ENSMUSG00000031278 | X | Acsl4 | Acyl-CoA synthetase long-chain family member 4 | -1.29954 | 0.000762 | 0.042973 |
|  | ENSMUSG00000046402 | 9 | Rbp1 | Retinol binding protein 1, cellular | -1.94839 | 2.09E-08 | 1.55E-05 |
|  | ENSMUSG00000015568 | 8 | Lpl | Lipoprotein lipase | -2.73116 | 1.56E-05 | 0.002854 |
|  | ENSMUSG00000025190 | 19 | Got1 | Glutamic-oxaloacetic transaminase 1, soluble | -1.30683 | 7.23E-05 | 0.008859 |
|  | ENSMUSG00000021242 | 12 | Npc2 | NPC intracellular cholesterol transporter 2 | -1.26822 | 0.000168 | 0.015593 |
