# Supplementary material for: Hepatic WDR23 proteostasis mediates insulin homeostasis by regulating insulin-degrading enzyme capacity
Source: GeroScience. 2024 May 20;46(5):4461–78. doi: 10.1007/s11357-024-01196-y (PMC11336002; doi:10.1007/s11357-024-01196-y)
Supplement: Supplementary file 8 — Supplementary file8 (DOCX 15 KB) [file 11357_2024_1196_MOESM8_ESM.docx]

**Table S2. The KEGG pathway enrichment analysis of DEGs in *Wdr23*KO mice liver tissues compare to the WT control with the threshold of *P*≤0.05**

| **Term** | **Gene ID** | **Chr** | **Name** | **Description** | **Log2 Fold Change** | ***P*-value** | ***P*-adj** |
| --- | --- | --- | --- | --- | --- | --- | --- |
| **Insulin signaling pathway**  **Up-regulated** | | | | | | | |
|  | ENSMUSG00000041237 | 3 | Pklr | Pyruvate kinase liver and red blood cell | 1.842783 | 3.55E-10 | 5.68E-07 |
|  | ENSMUSG00000020538 | 11 | Srebf1 | Sterol regulatory element binding transcription factor 1 | 2.246853 | 2.76E-07 | 0.000145 |
|  | ENSMUSG00000041798 | 11 | Gck | Glucokinase | 1.839161 | 5.97E-06 | 0.00141 |
|  | ENSMUSG00000025153 | 11 | Fasn | Fatty acid synthase | 1.817651 | 0.000268 | 0.021806 |
|  | ENSMUSG00000034793 | 11 | G6pc3 | Glucose 6 phosphatase, catalytic, 3 | 1.298063 | 0.000421 | 0.030001 |
|  | ENSMUSG00000020538 | 11 | Srebf1 | Sterol regulatory element binding transcription factor 1 | 2.246853 | 2.76E-07 | 0.000145 |
|  | ENSMUSG00000022383 | 15 | Ppara | Peroxisome proliferator activated receptor alpha | 1.19511 | 0.001098 | 0.054689 |
|  | ENSMUSG00000028978 | 5 | Nos3 | Nitric oxide synthase 3, endothelial cell | 1.637867 | 0.00291 | 0.09853 |
| **MAPK signaling pathway**  **Up-regulated** | | | | | | | |
|  | ENSMUSG00000054252 | 5 | Fgfr3 | Fibroblast growth factor receptor 3 | 1.303898 | 0.000326 | 0.025083 |
| **FoxO signaling pathway**  **Up-regulated** | | | | | | | |
|  | ENSMUSG00000034793 | 11 | G6pc3 | Glucose 6 phosphatase, catalytic, 3 | 1.298063 | 0.000421 | 0.030001 |
| **Glycolysis / Gluconeogenesis**  **Up-regulated** | | | | | | | |
|  | ENSMUSG00000041237 | 3 | Pklr | Pyruvate kinase liver and red blood cell | 1.842783 | 3.55E-10 | 5.68E-07 |
|  | ENSMUSG00000041798 | 11 | Gck | Glucokinase | 1.839161 | 5.97E-06 | 0.00141 |
|  | ENSMUSG00000034793 | 11 | G6pc3 | Glucose 6 phosphatase, catalytic, 3 | 1.298063 | 0.000421 | 0.030001 |
|  | ENSMUSG00000025236 | 9 | Adpgk | ADP-dependent glucokinase | 1.300949 | 0.00093 | 0.048996 |
| **Pyruvate metabolism**  **Up-regulated** | | | | | | | |
|  | ENSMUSG00000041237 | 3 | Pklr | Pyruvate kinase liver and red blood cell | 1.842783 | 3.55E-10 | 5.68E-07 |
|  |  |  |  |  |  |  |  |
| **PPAR signaling pathway**  **Up-regulated** | | | | | | | |
|  | ENSMUSG00000054422 | 6 | Fabp1 | Fatty acid binding protein 1, liver | 1.256687 | 0.000107 | 0.011728 |
|  | ENSMUSG00000010651 | 9 | Acaa1b | Acetyl-Coenzyme A acyltransferase 1B | 0.952087 | 0.000938 | 0.049223 |
| **AGE-RAGE signaling pathway**  **Down-regulated** | | | | | | | |
|  | ENSMUSG00000027962 | 3 | Vcam1 | Vascular cell adhesion molecule 1 | -1.33177 | 2.8E-05 | 0.004321 |
| **Glutathione metabolism**  **Down-regulated** | | | | | | | |
|  | ENSMUSG00000038155 | 19 | Gstp2 | Glutathione S-transferase, pi 2 | -3.93441 | 3.93E-11 | 7.12E-08 |
|  | ENSMUSG00000004038 | 3 | Gstm3 | Glutathione S-transferase, mu 3 | -3.42511 | 0.000332 | 0.025253 |
|  | ENSMUSG00000018339 | 11 | Gpx3 | Glutathione peroxidase 3 | -1.63836 | 0.000341 | 0.025717 |
|  | ENSMUSG00000028597 | 4 | Gpx7 | Glutathione peroxidase 7 | -3.05796 | 0.000596 | 0.036628 |
|  | ENSMUSG00000031584 | 8 | Gsr | Glutathione reductase | -1.10922 | 0.000844 | 0.046274 |
