# Supplementary material for: Hepatic WDR23 proteostasis mediates insulin homeostasis by regulating insulin-degrading enzyme capacity
Source: GeroScience. 2024 May 20;46(5):4461–78. doi: 10.1007/s11357-024-01196-y (PMC11336002; doi:10.1007/s11357-024-01196-y)
Supplement: Supplementary file 9 — Supplementary file9 (DOCX 17 KB) [file 11357_2024_1196_MOESM9_ESM.docx]

**Table S3. Differential expression of targeted proteins in *Wdr23*KO mice liver tissues compare to the WT control with the threshold of *P*≤0.05**

| **Term** | **Name** | **Description** | **Log2 Fold Change** | ***P*-value** | ***P*-adj** |
| --- | --- | --- | --- | --- | --- |
| **Carbohydrate metabolism**  **Up-regulated** | | | | | |
|  | SNX4 | Sorting nexin-4 | 0.083229 | 1.059386 | 0.00123 |
|  | GTR8/SLC2A8 | Glucose transporter type 8 | 0.123433 | 1.089324 | 0.047594 |
|  | RET4/RBP4 | Retinol-binding protein 4 | 0.164573 | 1.120834 | 0.015476 |
|  | VPS39 | Vam6/Vps39-like protein | 0.239837 | 1.180859 | 0.03333 |
|  | IDE | Insulin-degrading enzyme | 0.49103 | 1.405448 | 0.003979 |
|  | PLIN2 | Perilipin-2 | 0.595647 | 1.51115 | 0.044223 |
|  | CBPB2 | Carboxypeptidase B2 | 0.122307 | 1.088474 | 0.044443 |
|  | GALE | UDP-glucose 4-epimerase | 0.202877 | 1.150991 | 0.002718 |
| **Carbohydrate metabolism**  **Down-regulated** | | | | | |
|  | HRG | Haeme-responsive gene (HRG)-1 | -0.6263 | 0.647837 | 0.020691 |
|  | NAGTC | Sodium-dependent glucose transporter 1C | -0.23073 | 0.852203 | 0.016698 |
|  | C2C2L | Transmembrane protein 24 | -0.14151 | 0.906567 | 0.03487 |
|  | ACVR1/TGF | TGF-B superfamily receptor type I | -0.13201 | 0.91256 | 0.027382 |
|  | LMAN1 | Lectin mannose-binding 1 | -0.12298 | 0.918291 | 0.013709 |
|  | RAB10 | Ras-related protein Rab-10 | -0.08957 | 0.939802 | 0.016464 |
|  | XDH | Xanthine dehydrogenase | -0.11324 | 0.92451 | 0.004215 |
| **Insulin signaling**  **Up-regulated** | | | | | |
|  | PEDF | Serpin F1 | 0.078545 | 1.055952 | 0.021617 |
|  | SORCN | Sorcin | 0.174471 | 1.128551 | 0.023325 |
| **Insulin signaling**  **Down-regulated** | | | | | |
|  | LMAN2 | Lectin mannose-binding 2 | -0.19273 | 0.874951 | 0.036281 |
|  | BAIP2/IRSp53 | Insulin receptor substrate protein of 53 kDa | -0.13084 | 0.913297 | 0.001019 |
|  | OGT1 | O-GlcNAc transferase subunit p110 | -0.09375 | 0.937081 | 0.012789 |
| **PI3K/AKT/mTOR signaling**  **Up-regulated** | | | | | |
|  | MTOR | Serine/threonine-protein kinase mTOR | 0.071131 | 1.05054 | 0.010672 |
|  | LTOR1 | Late endosomal/lysosomal adaptor and MAPK and MTOR activator 1 | 0.08185 | 1.058374 | 0.049572 |
|  | GRP75 | Glucose-regulated protein 75 (GRP75), Heat shock protein 70 (HSP70) | 0.091203 | 1.065258 | 0.021406 |
|  | 1433Z | Protein kinase C inhibitor protein 1 | 0.135461 | 1.098444 | 0.015504 |
|  | ASNS | Glutamine-dependent asparagine synthetase | 1.777318 | 3.427883 | 0.022428 |
|  |  |  |  |  |  |
| **PI3K/AKT/mTOR signaling**  **Down-regulated** | | | | | |
|  | SCLY | Selenocysteine lyase | -0.21566 | 0.861155 | 0.020388 |
|  | NRN1 | Neuritin | -0.40308 | 0.756242 | 0.004372 |
|  | AKTS1/PRAS | Proline-rich AKT1 substrate 1 | -0.12297 | 0.918296 | 0.015158 |
| **FoxO signaling**  **Up-regulated** | | | | | |
|  | FCOR | Foxo1-corepressor | 0.421892 | 1.339683 | 0.000777 |
|  |  |  |  |  |  |
| **FoxO signaling**  **Down-regulated** | | | | | |
|  | HMGA1 | High mobility group protein HMG-I | -0.33595 | 0.792261 | 0.003751 |
| **MAPK signaling**  **Up-regulated** | | | | | |
|  | MK09 | MAPK9 | 0.110373 | 1.079507 | 0.000505 |
|  | ECSIT | SITPEC, Evolutionarily conserved signaling intermediate in Toll pathway | 0.115942 | 1.083682 | 0.025137 |
| **MAPK signaling**  **Down-regulated** | | | | | |
|  | M3K3 | MAPK/ERK kinase kinase 3 | -0.6179 | 0.651617 | 0.032129 |
|  | PAXI | Paxillin | -0.13104 | 0.91317 | 0.002378 |
|  | GAB1 | GRB2-associated-binding protein 1 | -0.07947 | 0.946408 | 0.006037 |
|  | SASH1 | SAM and SH3 domain-containing protein 1 | -0.11865 | 0.921048 | 0.027416 |
| **AMPK signaling**  **Down-regulated** | | | | | |
|  | CRBN | Cereblon | -0.93098 | 0.524503 | 0.010892 |
|  | PARP1 | Poly [ADP-ribose] polymerase 1 | -0.08163 | 0.94499 | 0.029268 |
|  | TBK1 | TANK-binding kinase 1 | -0.47323 | 0.720352 | 0.037901 |
| **Lipid metabolism**  **Up-regulated** | | | | | |
|  | ASAH1 | Acid ceramidase | 0.246443 | 1.186279 | 0.04491 |
|  | THIKB | Beta-ketothiolase B | 0.349508 | 1.274126 | 0.04113 |
| **Lipid metabolism**  **Down-regulated** | | | | | |
|  | ELOV1 | Elongation of very long chain fatty acids protein 1 | -0.441 | 0.736622 | 0.010616 |
|  | AAKG2 | AMP-activated protein kinase subunit gamma-2 | -0.43151 | 0.741486 | 0.045831 |
|  | OSBL2 | Oxysterol-binding protein-related protein 2 | -0.32967 | 0.795717 | 0.027604 |
| **Oxidation-reduction**  **Up-regulation** | | | | | |
|  | NDUF5 | NADH dehydrogenase [ubiquinone] 1 alpha subcomplex assembly factor 5 | 0.28191 | 1.215804 | 0.027516 |
|  | NNTM | Nicotinamide nucleotide transhydrogenase | 2.126628 | 4.366955 | 5.49E-06 |
|  | TMLH | Trimethyllysine dioxygenase | 0.320616 | 1.248864 | 0.014325 |
| **Oxidation-reduction**  **Down-regulation** | | | | | |
|  | COX19 | Cytochrome c oxidase assembly protein COX19 | -0.33592 | 0.792279 | 0.008761 |
|  | AOFA | Amine oxidase [flavin-containing] A | -0.33583 | 0.79233 | 0.047069 |
