# Supplementary material for: Hepatic WDR23 proteostasis mediates insulin homeostasis by regulating insulin-degrading enzyme capacity
Source: GeroScience. 2024 May 20;46(5):4461–78. doi: 10.1007/s11357-024-01196-y (PMC11336002; doi:10.1007/s11357-024-01196-y)
Supplement: Supplementary file 10 — Supplementary file10 (DOCX 31 KB) [file 11357_2024_1196_MOESM10_ESM.docx]

| **Protein** | **Ratio (KO/WT)** | **p-value** | **Description** | **Role** |
| --- | --- | --- | --- | --- |
| YWHAZ | 1.098443735 | 0.015503955 | 14-3-3 protein zeta/delta | Glucose Metabolism^1^ |
| FCOR | 1.339683216 | 0.00077717 | Foxo1-corepressor and cellular glucose homeostasis | Transcriptional regulation ^2^ |
| AAKG2 | 0.741485544 | 0.045830957 | AMP/ATP-binding subunit of AMP-activated protein kinase (AMPK), an energy sensor protein kinase that plays a key role in regulating cellular energy metabolism. | Glucose metabolism^3^ |
| IMPA2 | 1.141395562 | 0.000643951 | Inositol monophosphatase 2 | Metabolic signaling^4^ |
| PGP | 1.095449769 | 0.039508474 | Phosphoglycolate phosphatase | Healthy aging ^5^ |
| MTOR | 1.05054021 | 0.010672401 | Mechanistic target of rapamycin kinase | Metabolic signaling^6^ |
| CBP2 | 1.088474324 | 0.044443196 | Prohormone processing enzyme; carboxypeptidase | Metabolic homeostasis^7^ |
| SLC2A8 | 1.089323636 | 0.047593704 | Solute carrier family 2, (facilitated glucose transporter), member 8 | Carbohydrate transporter^8^ |
| GALE | 1.150991471 | 0.00271846 | UDP-glucose 4-epimerase | Carbohydrate metabolism^9^ |
| ASNS | 3.42788289 | 0.022427988 | Asparagine synthetase | Response to glucose restriction |
| IDE | 1.405447674 | 0.003978549 | Insulin degrading enzyme | Insulin degradation^10,11^ |

**Table S4. ChIP-X Enrichment Analysis 3 (ChEA3) of transcription factors in *Wdr23*KO mice liver tissues compare to the WT (C57BL/6J) control with the threshold of p≤0.05.**
