# Supplementary material for: Hepatic WDR23 proteostasis mediates insulin homeostasis by regulating insulin-degrading enzyme capacity
Source: GeroScience. 2024 May 20;46(5):4461–78. doi: 10.1007/s11357-024-01196-y (PMC11336002; doi:10.1007/s11357-024-01196-y)
Supplement: Supplementary file 11 — Supplementary file11 (DOCX 16 KB) [file 11357_2024_1196_MOESM11_ESM.docx]

**Table S5: The multiple comparisons of HepG2 cells treated with insulin (Figure 4)/IDE inhibitor (ML345) (Figure 5) /siRNA (Figure 6).** *p<.05, **p<.01, ***p<.001, ****p<.0001

|  | **WT** | ***WDR23(-/-)*** | **WT+Insulin** | ***WDR23(-/-)+*Insulin** |
| --- | --- | --- | --- | --- |
| **4B pIRS1/IRS1** |  |  |  |  |
| AVE | 1 | 1.129752333 | 1.839634333 | 1.73286475 |
| STD | 0.076085679 | 0.256439072 | 0.576232771 | 0.592404259 |
| vs WT |  | NS | * (0.0119) | * (0.0149) |
| vs WT+Insulin | * (0.0119) | * (0.0370) |  | NS |
| vs *WDR23(-/-)* | NS |  | *(0.0370) | NS |
|  |  |  |  |  |
| **4C pAKT2/AKT2** |  |  |  |  |
| AVE | 1.000000333 | 3.337181167 | 2.17236 | 3.401331333 |
| STD | 0.290462397 | 0.823420221 | 0.586693781 | 0.587746182 |
| vs WT |  | **** (<0.0001) | *(0.0178) | ***(0.0002) |
| vs WT+Insulin | *(0.0178) | *(0.0186) |  | *(0.0472) |
| vs *WDR23(-/-)* | **** (<0.0001) |  | *(0.0186) | NS |
|  |  |  |  |  |
| **4D pMAPK/MPAK** |  |  |  |  |
| AVE | 1 | 2.104522833 | 0.426228333 | 0.817758333 |
| STD | 0.147963167 | 0.151615086 | 0.032320735 | 0.156764845 |
| vs WT |  | **** (<0.0001) | **** (<0.0001) | NS |
| vs WT+Insulin | **** (<0.0001) | **** (<0.0001) |  | *** (0.0003) |
| vs *WDR23(-/-)* | **** (<0.0001) |  | **** (<0.0001) | **** (<0.0001) |
|  |  |  |  |  |
| **4E pmTOR/mTOR** |  |  |  |  |
| AVE | 1 | 0.501111833 | 1.232476 | 1.0614565 |
| STD | 0.385992571 | 0.259033056 | 0.684076804 | 0.459314501 |
| vs WT |  | *(0.0295) | NS | NS |
| vs WT+Insulin | NS | NS |  | NS |
| vs WDR23(-/-) | *(0.0295) |  | *(0.0438) | * (0.0264) |
|  |  |  |  |  |
| **4F pFoxO/FoxO** |  |  |  |  |
| AVE | 1 | 1.6089285 | 1.689140667 | 2.12822 |
| STD | 0.309924536 | 0.333117826 | 0.311910214 | 0.354368427 |
| vs WT |  | *(0.0447) | **(0.0091) | **** (<0.0001) |
| vs WT+Insulin | **(0.0091) | NS |  | NS |
| vs *WDR23(-/-)* | *(0.0447) |  | NS | *(0.0488) |
|  |  |  |  |  |

|  | **WT** | ***WDR23(-/-)*** | **WT+ML345** | ***WDR23(-/-)+*ML345** |
| --- | --- | --- | --- | --- |
| **5B pIRS1/IRS1** |  |  |  |  |
| AVE | 1 | 1.129752333 | 0.656455167 | 0.7023165 |
| STD | 0.076085679 | 0.256439072 | 0.265420431 | 0.22245872 |
| vs WT |  | NS | NS | NS |
| vs WT+ML345 | NS | **(0.0064) |  | NS |
| vs *WDR23(-/-)* | NS |  | **(0.0064) | NS |
|  |  |  |  |  |
| **5C pAKT2/AKT2** |  |  |  |  |
| AVE | 1.000000333 | 3.337181167 | 4.917812667 | 1.577501333 |
| STD | 0.290462397 | 0.823420221 | 1.676179433 | 0.235925309 |
| vs WT |  | **(0.0020) | **** (<0.0001) | ** (0.0036) |
| vs WT+ML345 | **** (<0.0001) | *(0.0428) |  | **** (<0.0001) |
| vs *WDR23(-/-)* | **(0.0020) |  | *(0.0428) | *(0.0215) |
|  |  |  |  |  |
| **5D pMAPK/MPAK** |  |  |  |  |
| AVE | 1 | 2.104522833 | 0.890527333 | 0.003725167 |
| STD | 0.147963167 | 0.151615086 | 0.173993993 | 0.003071636 |
| vs WT |  | **** (<0.0001) | NS | **** (<0.0001) |
| vs WT+ML345 | NS | **** (<0.0001) |  | **** (<0.0001) |
| vs *WDR23(-/-)* | **** (<0.0001) |  | **** (<0.0001) | **** (<0.0001) |
|  |  |  |  |  |
| **5E pmTOR/mTOR** |  |  |  |  |
| AVE | 1 | 0.501111833 | 0.421904667 | 0.239807833 |
| STD | 0.385992571 | 0.259033056 | 0.062450416 | 0.029157083 |
| vs WT |  | **(0.0075) | **(0.0020) | **** (<0.0001) |
| vs WT+ML345 | **(0.0020) | NS |  | NS |
| vs WDR23(-/-) | **(0.0075) |  | NS | NS |
|  |  |  |  |  |
| **5F pFoxO/FoxO** |  |  |  |  |
| AVE | 0.999999833 | 1.6089285 | 0.767191167 | 0.060154 |
| STD | 0.309924536 | 0.333117826 | 0.175445056 | 0.049561056 |
| vs WT |  | **(0.0039) | NS | **** (<0.0001) |
| vs WT+ML345 | NS | ***(0.0001) |  | ***(0.0003) |
| vs *WDR23(-/-)* | **(0.0039) |  | ***(0.0001) | **** (<0.0001) |
|  |  |  |  |  |

|  | **WT** | ***WDR23(-/-)*** | ***WDR23(-/-)* + siCT** | ***WDR23(-/-)* + siNRF2** |
| --- | --- | --- | --- | --- |
| **6B_IDE mRNA** |  |  |  |  |
| AVE | 1 | 1.276780667 | 1.0786925 | 0.668673556 |
| STD | 0.010540926 | 0.177479592 | 0.19214541 | 0.073146078 |
| vs WT |  | ***(0.0008) | NS | **** (<0.0001) |
| vs *WDR23(-/-) +* siCT | NS | NS |  | ***(0.0001) |
| vs *WDR23(-/-)* | ***(0.0008) |  | NS | **** (<0.0001) |
|  |  |  |  |  |
| **6C_IDE protein** |  |  |  |  |
| AVE | 1.000000125 | 1.988318729 | 1.796724179 | 0.87327132 |
| STD | 0.4614295 | 0.749699239 | 0.702777822 | 0.516617918 |
| vs WT |  | ***(0.0009) | *(0.0178) | *(0.0464) |
| vs WDR23(-/-) + siCT | *(0.0178) | NS |  | *** (0.0001) |
| vs WDR23(-/-) | ***(0.0009) |  | NS | ***(0.0009) |
|  |  |  |  |  |
